# Immunomodulatory Effect of *Agave tequilana* Evaluated on an Autoimmunity Like-SLE Model Induced in Balb/c Mice with Pristane

**DOI:** 10.3390/molecules22060848

**Published:** 2017-05-25

**Authors:** Zúlima Jannette Gutiérrez Nava, Antonio Ruperto Jiménez-Aparicio, Maribel Lucila Herrera-Ruiz, Enrique Jiménez-Ferrer

**Affiliations:** 1 Centro de Desarrollo de Productos Bióticos (CEPROBI), Instituto Politécnico Nacional (IPN), Yautepec, Morelos C.P. 62731, Mexico; zjgn06@gmail.com; 2 Centro de Investigación Biomédica del Sur (CIBIS), Instituto Mexicano del Seguro Social (IMSS), Xochitepec, Morelos C.P. 62790, Mexico; arjaparicio@gmail.com (A.R.J.-A.); cibis_herj@yahoo.com.mx (M.L.H.-R.)

**Keywords:** *Agave tequilana*, inflammation, autoimmunity, SLE, pristane

## Abstract

In this work, the immunomodulatory activity of the acetone extract and the fructans obtained from *Agave tequilana* were evaluated, on the systemic autoimmunity type-SLE model generated by the administration of 2,6,10,14-tetramethylpentadecane (TMPD, also known as pristane) on Balb/c female mice. The systemic autoimmunity type-SLE was observed seven months after the application of TMPD, in which the animals from the negative control group (animals with damage and without any other treatment) developed articular inflammation, proteinuria, an increment of the antinuclear antibody titters and tissue pro-inflammatory cytokines levels (IL-1β, IL-6, TNF-α e IFN-γ) as well as the anti-inflammatory cytokine IL-10. The administration of the different treatments and the extracts of *A. tequilana*, provoked the decrease of: articular inflammation, the development of proteinuria, ssDNA/dsDNA antinuclear antibody titters and cytokines IL-1β, IL-6, TNF-α, IFN-γ and IL-10. The phytochemical analysis of the acetone extract identified the presence of the following compounds: β-sitosterol glycoside; 3,7,11,15-tetramethyl-2-hexadecen-1-ol (phytol); octadecadienoic acid-2,3-dihydroxypropyl ester; stigmasta-3,5-dien-7-one; cycloartenone and cycloartenol. Therefore, *A. tequilana* contains active compounds with the capacity to modify the evolution of the systemic autoimmunity type-SLE on a murine model.

## 1. Introduction

Systemic lupus erythematosus (SLE), is a chronic-inflammatory autoimmune ilness that is characterized by producing autoantibodies that recognize different subcellular components, organs and systems of the host, like DNA, kidneys, skin, joints, etc. [1]. In accordance to the American College of Rheumatology, SLE is 10 times more frequent in women than in men. Due to the complexity of this illness, the treatment of SLE is based on the combined administration of different drugs, which include among others glucocorticoids, non-steroidal anti-inflammatory drugs (NSAIDs), cyclooxygenase-2 anti-inflammatory inhibitors (COXIBs), antimalarials and immunosuppressants. Nevertheless, most of these treatments cause adverse effects that include osteoporosis, hepatotoxicity, kidney damage, susceptibility to infections, among others, which generates a general health deterioration in patients [2], which represents a challenge in the search for alternative treatments; therefore, medicinal plants offer a good source for the development of potential phytopharmaceuticals with anti-inflammatory and immunomodulatory activity.

The genus *Agave* is endemic of America, confirmed by the presence of approximately 200 species, of which 75% are found in Mexico [3]. Evidence of pharmacological activity has been reported for this genus, where anti-inflammatory, antiparasitic, cytotoxic, anticancer and immunomodulatory effects are some of the most important [4,5,6]. *Agave tequilana* (blue agave) is a Mexican plant with an important commercial value, since the raw material is used for the production of tequila. In previous studies of *A. tequilana* pharmacological activity has been proven; on the one hand, fructans obtained from this plant decreased the serum levels of cholesterol and triglycerides and increased the count of fecal bifidobacteria in humans [7,8]. A diet rich in *A. tequilana* fiber (ADF) reduces the serum levels of malondialdehyde and decreases oxidative stress in hypercolesterolemic rats [9]; also, an ADF mixture with *Hibiscus sabdariffa* increased the plasmatic levels of glucagon-like peptide-1 (GLP1) and reduced the pro-inflammatory cytokines and increased IL-10 levels in adipose tissue of obese mice [10]. Furthermore, the acetone extract of *A. tequilana* leaves has anti-inflammatory capacity tested on the inflammatory mice ear oedema model using phorbol 12-myristate 13-acetate (TPA) [11]. Up to now, the pharmacological potential of *Agave* fructans has been well established [8,12]. In contrast, only one published report mentions the anti-inflammatory capacity of the acetone extract of *A. tequilana* leaves, however, the compound responsible for this activity was not revealed [11]. Other studies have identified three homoisoflavones present in the acetone extract of *A. tequilana* but no biological activity has been proven [13]; therefore, the present work represents the first study where the immunomodulatory activity is evaluated and active compounds of the extract of leaves of *A. tequilana* were identified.

The aim of the present work was to determine the immunomodulatory activity of *A. tequilana* on an experimental autoimmune type-SLE model induced by the administration of 2,6,10,14-tetramethylpentadecane (TMPD, pristane) to female Balb/c mice.

## 2. Results

### 2.1. Extracts and Standardized Fractions from Agave tequilana

The acetone extract yield from the dried plant was 1.07%. Standardization of the acetone extract composition was done through a gas chromatographic-mass spectrometry analysis, which indicated the presence of the following compounds: (yields presented in mg/g of extract): β-sitosterol glucoside (0.1995 mg/g); 3,7,11,15-tetramethyl-2-hexadecen-1-ol (phytol, 0.1615 mg/g); octadecadienoic acid 2,3-dihydroxypropyl ester (0.114 mg/g); stigmasta-3,5-dien-7-one (0.0285 mg/g); cycloartenol (0.019 mg/g); *n*-hexadecanoic acid (0.304 mg/g); octadecanoic acid (0.494 mg/g) (Table 1).

### 2.2. Experimental Autoimmune Type-SLE Model, Joint (Knee) Swelling and Albuminuira

**Figure 1:**
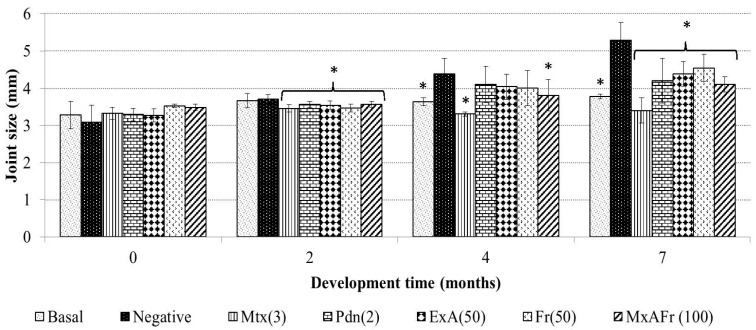
Effect of the oral administration of different treatments on joint size of Balb/c female mice with SLE induced with pristane. Methotrexate, 3 mg/Kg (Mtx); prednisone, 2.5 mg/Kg (Pdn); *Agave* acetone extract, 50 mg/Kg (ExA); fructans, 50 mg/Kg (Fr); mixture of acetone extract and fructans, 100 mg/Kg (MxAFr) (each column represents an average ± S.E., *n* = 12, ANOVA, Bonferroni post-test, * *p* < 0.05 compared to the negative control group).

Long term administration of TMPD generated a significant increment of the articular diameter of the animals that were exposed to the SLE model. In Figure 1, it can be appreciated that after seven months the negative control group presented a 43.8% relative increment of their articular joints; the relative value was applied since the determinations were done considering the increase of joint diameter associated to the mice corporal growth. The relative increment of the articular diameter was statistically significant and it was evident until the fourth month post-administration of TMPD (* *p* < 0.05). When comparing the articular diameters of the negative control group with those of methotrexate and prednisone, it was observed that both blocked the increase of joint inflammation caused by TMPD (* *p* < 0.05). The comparative analysis of the groups that received the *A. tequilana* treatments against the negative control group showed a statistically significant decrease of the articular diameter (* *p* < 0.05).

**Figure 2:**
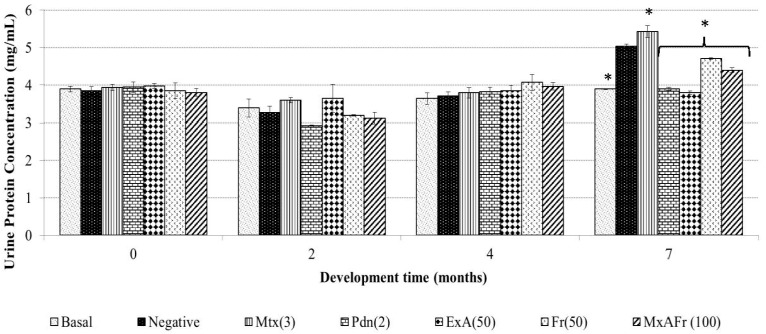
Effect of oral administration of different treatments on the concentration of urine protein of Balb/c female mice with SLE induced with pristane. Methotrexate, 3 mg/Kg (Mtx); prednisone, 2.5 mg/Kg (Pdn); *Agave* acetone extract, 50 mg/Kg (ExA); fructans, 50 mg/Kg (Fr); mixture of acetone extract and fructans, 100 mg/Kg (MxAFr) (each column represents an average ± S.E., *n* = 12, ANOVA, Bonferroni post-test, * *p* < 0.05 compared to the negative control group).

Another cardinal SLE symptom is the establishment of lupus nephritis secondary to immune complex deposits and cellular infiltration in the glomerulus, as well as in the nephron vasculature or in the tubular segment of said structure [13]. Proteinuria was evident seven months after TMPD was administered (Figure 2) as TMPD generated a 31% increase in proteinuria that was statistically significant with respect to the basal control group (* *p* < 0.05).

The treatment with Mtx increased proteinuria in 41%, which was statistically significant respectively to the basal control (* *p* < 0.05); the increment in proteinuria with Mtx was even higher than the one with TMPD treatment (31%), since Mtx has a significant nephrotoxic effect [14]. Pnd treated animals maintained protein level the same as the basal control group with no statistically significant difference. The administration of both the *Agave tequilana* ExA extract as well as the Fr and MxAFr fractions significantly decreased the levels of proteinuria associated with SLE induced by TMPD.

### 2.3. Autoimmune Response in Type-SLE Model

**Figure 3:**
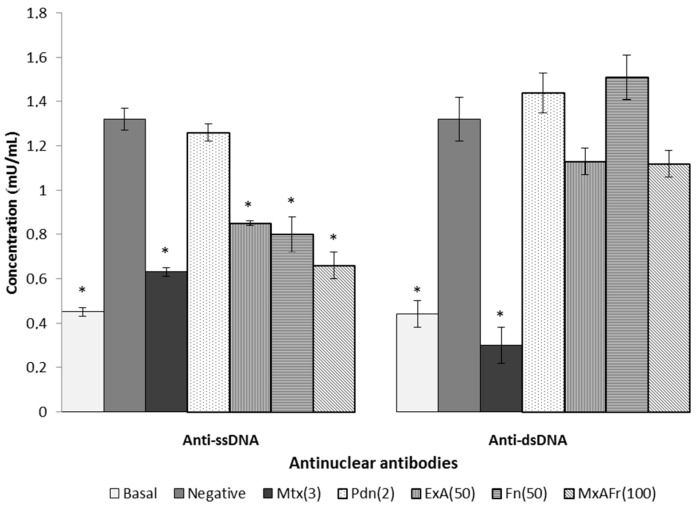
Effect of oral administration of different treatments on the concentration of antinuclear autoantibodies (anti-ssDNA, anti-dsDNA) in serum of Balb/c female mice with SLE induced by pristine administration. Methotrexate, 3 mg/Kg (Mtx); prednisone, 2.5 mg/Kg (Pdn); *Agave* acetone extract, 50 mg/Kg (ExA); fructans, 50 mg/Kg (Fr); mixture of acetone extract and fructans, 100 mg/Kg (MxAFr) (each column represents an average ± S.E., *n* = 12, ANOVA, Bonferroni post-test, * *p* < 0.05 compared to the negative control group).

Another established criterion for the diagnosis of SLE is the autoimmune response defined by the appearance of anti-ssDNA and anti-dsDNA autoantibodies. The administration of BALB/c mice with TMPD induced the production of anti-ssDNA and anti-dsDNA autoantibodies [15]. This is shown in Figure 3, where the negative control group had 3.5 times higher titers of both autoantibodies, compared to the basal control group (*p* < 0.05). Mtx treatment produced a statistically significant decrease of both autoantibody titers compared to the negative control (* *p* < 0.05). However, Pdn did not produce any difference in anti-ssDNA and anti-dsDNA titers compared to the negative control group. On the other hand, all of *A. tequilana* treatments produced a statistically significant reduction (* *p* < 0.05) of the anti-ssDNA autoantibody titers; whereas the ExA and MxAFr treatments presented a tendency to decrease the levels of anti-dsDNA autoantibody titers. The autoimmune response in animal models that develop SLE is evident when the levels of antinuclear autoantibodies are elevated. TMPD’s capacity to affect the model SLE type autoimmunity is confirmed with the increase of anti-ssDNA and anti-dsDNA autoantibodies like in the negative control group (compared to the basal group); therefore, the increase in the autoantibody titers in animals exposed to TMPD indicates a development of a reactive process of the immune system against their own antigens [15]. The three *A. tequilana* treatments decreased the anti-ssDNA antinuclear autoantibody titers compared to the negative control. Meanwhile, only ExA and MxAFr decreased the anti-dsDNA antinuclear autoantibody titers, but without presenting a statistically significant decrease of this parameter compared to the negative control.

### 2.4. Cytokines Response in Type-SLE Model

**Figure 4:**
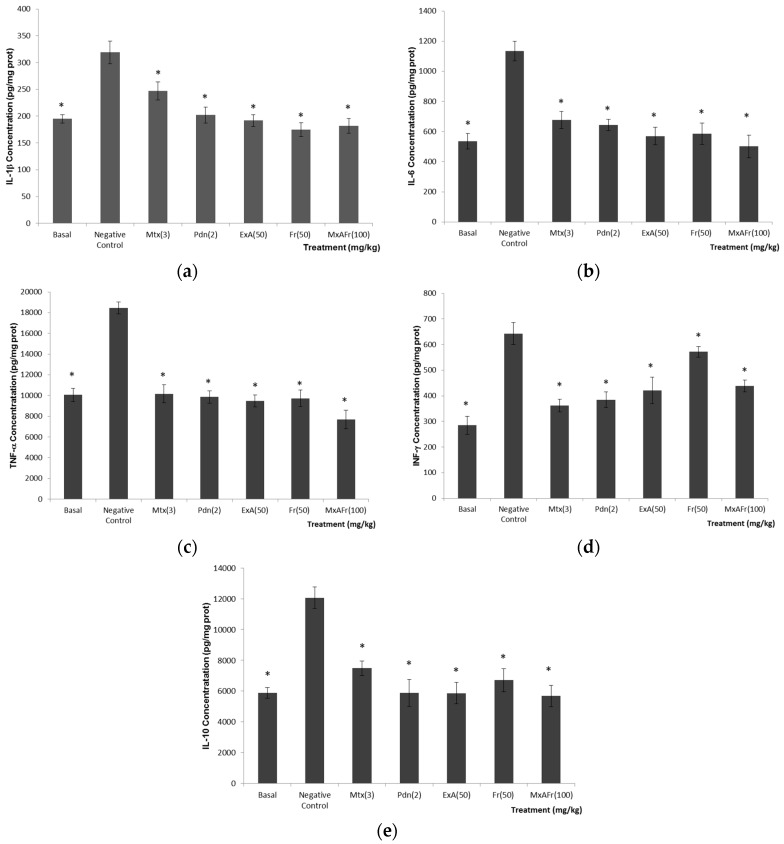
Effect of oral administration of different treatment on the concentrations of different cytokines obtained from kidney homogenates of Balb/c female mice with SLE induced by TMPD. (**a**) IL-1β; (**b**) IL-6; (**c**) TNF-α; (**d**) IFN-γ; (**e**) IL-10. Methotrexate, 3 mg/Kg (Mtx); prednisone, 2.5 mg/Kg (Pdn); *Agave* acetone extract, 50 mg/Kg (ExA); fructans, 50 mg/Kg (Fr); mixture of acetone extract and fructans, 100 mg/Kg (MxAFr) (each bar represents an average ± S.E., *n* = 12, ANOVA, Bonferroni post-test, * *p* < 0.05 compared to the negative control group).

The results of the tissue concentration of proinflammatory (IL-1β, IL-6, TNF-α, INF-γ) and anti-inflammatory (IL-10) cytokines are shown in Figure 4(a–e) respectively. The TMPD negative control group showed a statistically significant increase in the concentration of pro- and anti-inflammatory cytokines compared to the basal group (* *p* < 0.05). Both anti-inflammatory positive control groups (Mtx and Pdn) inhibited the production of pro and anti-inflammatory cytokines when compared with the negative control group (* *p* < 0.05). All the treatments derivate from *A. tequilana* achieved a statistically significant decrease of the concentrations of proinflammatory cytokines IL-1β, Il-6, TNF-α y IFN-γ and of anti-inflammatory cytokine IL-10 when compared to the negative control group (* *p* < 0.05).

## 3. Discussion

Intraperitoneal administration of TMPD, an isoprenoid oil, generates glomerulonephritis, arthritis, autoimmunity, systemic inflammation, among others; therefore, it is considered a good SLE model since it develops at least four of the 11 established criteria of the human pathology. The mechanism of action by which TMPD induces arthritis consists of neutrophil and mononuclear cell infiltration into the joint cavity, as well as the development of synovial hyperplasia by pannus formation, at the same time there is a raise of mRNA relative to the increase of the protein expression of cytokines like TNF-α, IL-1β and IL-6, the increase of seric levels of rheumatoid factor and autoantibodies against type I and II collagen and complemented by the augmentation of the articular joints [15,16].

In the present work, it was established that the administration of TMPD generated an increment of the joint size of the treated mice, secondary to an articular inflammation process. The anti-inflammatory effect of Mtx can be explained by the mechanism of action of this drug, Mtx is an antimetabolite that inhibits folic acid conversion to tetrahydrofolate, suppressing in an unspecific way cellular proliferation; therefore, it presents an immunosupressor effect, which modifies immune cell proliferation [17]. This drug is wildly used in the Mexican public health care system in highly specialized clinical treatment of SLE; Mtx is given to patients with persistent symptoms of this illness that generally present tissular damage of different organs and articular destruction and where corticosteroid treatments were not effective [18]. Furthermore, seven months after TMPD administration the corticosteroid prednisone also decreased the diameter of the joints due to the anti-inflammatory capacity of its active form denominated prednisolone, which stimulates the production of inhibitor lipoproteins of phospholipase A2 blocking arachidonic acid synthesis, thus, eicosanoid production, this anti-arthritic effect of prednisone coincides with what has already been reported [16].

Meanwhile, the decrease of the articular diameter caused by the administration of the extracts of *A. tequilana* can be associated to the presence of active secondary metabolites like β-sitosterol glycoside, 3,7,11,15-tetramethyl-2-hexadecen-1-ol (phytol), octadecadienoic acid 2,3-dihydroxy-propyl ester, stigmasta-3,5-dien-7-one, cycloartenone, cycloartenol and fructans. β-Sitosterol glycoside, phytol, cycloartenone and cycloartenol isolated from *Agave tequilana* have been reported to present anti-inflammatory and immunomodulatory capacity in different inflammatory studies [19,20,21,22] and the articular anti-inflammatory effect of the fructans from *A. tequilana* could be due to the fact fructans participate in the activation of toll-like receptors type 2 and 4 (TLR 2and TLR4) that have also been related to have immunomodulatory and anti-inflammatory capacity which is independent to the prebiotic effect of said molecules [12].

Proteinuria determination for the ExA treatment presented the same effect than Pdn as well as to the basal group with no statistically significant difference. The other two treatments of Fr and MxAFr, were not as efficient in the inhibition of the proteinuria associated to the SLE secondary to TMPD administration, even though they were statistically different from the negative control and the Mtx groups. Proteinuria is one of the principal components of lupus nephritis [23]. It is generated as a result of the damage to the nephron as a consequence of chronic inflammation caused by the accumulation of immune complexes, which has been widely documented in the SLE model induced on BALB/c mice by TMPD administration [15]. Said complexes are constituted by IgG and components of the complement system; the damage to the nephron alters the filtration process and glomerular reabsorption. The alteration of the glomerular basements membrane and the endothelium modify the filtration process, therefore, the proteins pass through to the tubular space of the nephron and then are eliminated in the urine [24]. In the model used, proteinuria development was not observed four months after TMPD was administered as reported by [25]; although, after seven months an increment of albumin elimination in urine was registered. Mtx was capable of controlling the inflammatory process as observed in Figure 1; it also provoked an increment of protein elimination in urine; this represents one of the collateral effects previously reported for the strain of mice used in this model [26] and this is what determines the deleterious capacity of Mtx on the nephron. The anti-inflammatory effect of Pdn also protected the nephrons from damage since proteinuria was not provoked, as has previously been reported in laboratory tests and clinical studies [27,28], where it has been suggested that Pdn has the capacity to improve the nephron function. 

All the treatments from *A. tequilana* had the capacity to decrease proteinuria levels with a statistically significant difference compared to the negative control group; the most effective treatment was ExA, since it did not present a statistical difference when compared to the positive control Pdn and the basal group. Also, it can be considered that the observed effect with these treatments are associated to the presence of the following compounds: β-sitosterol glycoside, phytol, octadecadienoic acid 2,3-dihydroxypropyl ester, stigmasta-3,5-dien-7-one, cycloartenone and cycloartenol, that have already been proven to have anti-inflammatory and immunomodulatory activity [19,20,21,22].

Regarding the typical autoimmune response of SLE defined by the appearance of anti-ssDNA and anti-dsDNA autoantibodies it can be considered that prolonging the time and/or increasing the dose of these two treatments could result in the significant reduction of the anti-dsDNA antinuclear autoantibodies titers. Mtx’s capacity to decrease anti-ssDNA and anti-dsDNA titers can be a consequence of lymphocyte apoptosis activated in phase S/G2 of the cell cycle that is induced by the presence of this drug, resulting in the immunosuppression of antinuclear autoantibodies [29]. Pdn managed to reduce articular inflammation of the joints caused by TMPD, however, was not able to control the increase in antinuclear autoantibodies titers. Some reports indicate that daily intragastric administration of Pdn at a dose of 2.5 mg/kg for three weeks blocked the increase of antinuclear autoantibodies titers associated with SLE development in MRL/lpr mice [28]; the Pdn treatment used in this assay did not have the capacity to modify the antinuclear autoimmune response, it can be considered that increasing the dose of this drug up to 5 mg/kg in this same treatment, it could generate a decrease in the antinuclear autoantibodies titers.

Once again it is suggested that the participation of compounds such as β-sitosterol glycoside, phytol, octadecadienoic acid 2,3-dihydroxypropyl ester, stigmasta-3,5-dien-7-one, cycloartenone and cycloartenol and fructanes, can be responsible for modulating the Th2 type immune response, characteristic of SLE secondary to the administration of TMPD, confirming that such compounds have the capacity to generate an immunomodulatory effect [19,20,21,22].

Modulation of the antinuclear autoimmune response present in SLE depends on different factors, where antigen reactivity is the most important one, therefore, in the evaluation of antigens it can be considered that ssDNA reactivity is due to the exposure of the antibodies to the non-polar components of the nucleic acids and also of the phosphate groups, consequently making the molecule a low potency immunogen since it is less reactive. In contrast to dsDNA, it is an antigen of greater immunogenic potency, because it exposes the electrical charges of ionized phosphate groups in the double strand of DNA. This may explain why the treatments with the *Agave* extracts were more effective against the production of antinuclear antibodies, directed against ssDNA, which corresponds to the antigen of lower antigenic capacity. 

There are reports where the development of autoimmunity, arthritis, glomerulonephritis, pulmonary vasculitis among others through the injection of TMPD, are the results of an increased production of cytokines like IL-6, IL-1, IL-12, TNF-α, as well as, the IFN’s receptors that are responsible for releasing a chronic inflammation process [4,30,31,32].

IL-1β (Figure 4a), is a primary immune response activator that promotes inflammation; it is considered that IL-1RN receptors, which recognize this cytokine, play an important role in the systemic inflammation in arthritis development and kidney damage associated to SLE type autoimmunity [30,33,34,35]. IL-6 (Figure 4b), is a cytokine produced by different types of cells, and this cytokine is considered to participate in the differentiation of CD4+ T cells and in the production of B cells, which stimulate the production of autoantibodies. There are reports where the deficiency of IL-6 is related to the absence of the production of anti-ssDNA and anti-dsDNA autoantibodies in Balb/c mice with SLE induced with TMPD. Deficiency of IL-6 in MRL-Fas lpr mice delay lupus nephritis [30,36]. TNF-α (Figure 4c) is a proinflammatory cytokine produced by monocytes and macrophages; TNF-α participates in the regulation of lymphocyte apoptosis and consolidates the proinflammatory response by inducing the liberation of IL-1β among other functions [37]. The role that TNF-α plays in the development of SLE is unknown, since, some authors report that the treatment with drugs that inhibit this cytokine has a therapeutic effect in patients with SLE, but it is also reported that in some cases inhibition of this cytokine results in the increase in the severity of this illness [38]. Interferons (IFN’s) are cytokines that possess a pleiotropic effect and it is considered that through the union of their receptors and the activation of transcription (STAT) participate in autoimmune development [39]. IFN-γ (Figure 4d), is a cytokine produced by activated T cells, natural killers, macrophages and dendritic cells. According to [30], IFN-γ is considered an important cytokine that participates in the development of lupus nephritis and in the increase of anti-DNA/chromatin titers [30]. The last cytokine evaluated was IL-10 (Figure 4e); it is considered a key anti-inflammatory cytokine for controlling the extension and duration of the immune response. It participates as an important anti-inflammatory, selectively blocking proinflammatory gene expression that codifies for cytokines and chemokines; it also enhances the production and expression of anti-inflammatory molecules. Nevertheless, different studies indicate that blocking the expression of this cytokine results in the decrease immunoglobulin production controlling the illness development [38,40]. β-Sitosterol glycoside, 3,7,11,15-tetramethyl-2-hexadecen-1-ol (phytol), octadecadienoic acid 2,3-dihydroxypropyl ester, stigmasta-3,5-dien-7-one, cycloartenone and cycloartenol and fructans, could be the possible secondary metabolites isolated from *A. tequilana* leaves responsible for the anti-inflammatory properties of the administered extracts [19,20,21,22]. Anti-inflammatory effect would be one of the mechanisms by which the *A. tequilana* treatments would be acting as modulators in the development of SLE-type autoimmunity induced by the administration of TMPD.

## 4. Materials and Methods

### 4.1. Chemical Reagents and Drugs

2,6,10,14-Tetramethylpentadecane (TMPD, also known as pristane, synthetic, purity > 98%) was purchased from Sigma-Aldrich^®^ (Sigma-Aldrich Corporate, St. Louis, MO, USA). Methotrexate and prednisone were selected from the list of drugs provided by the Mexican Social Security Institute for the treatment of lupus erythematosus. Kits for the determination of cytokines, IL-1β, IL-6, TNF-α, INF-γ and IL-10 were purchased from BD Biosciences Inc. (Benton Dickinson and Company, San Diego, CA, USA). The determination of urine protein was done through two tests: semiquantitative analysis using Combur10 Test (Roche Diagnostics, Indianapolis, IN, USA) dipsticks purchased from Eypro SA de CV and by quantitative analysis by the Bradford reagent method, with reagents purchased from Sigma-Aldrich^®^. Reactive-grade solvents (acetone, ethanol, methanol, ethyl acetate and *n*-hexane) purchased from VWR International Corporate (VWR International Corporate, Radnor, PA, USA) were used in the chemical separation; the first two solvents were used to obtain the extracts of *A. tequilana*. The solvent *n*-hexane was used in the first fractionation of the acetone extract of *A. tequilana*, and ethyl acetate was used in a second fractionation of the acetone extract of *A. tequilana*, from where the following compounds were isolated: β-sitosterol glycoside, 3,7,11,15-tetramethyl-2-hexadecen-1-ol (phytol), octadecadienoic acid 2,3-dihydroxypropyl ester, stigmasta-3,5-dien-7-one, cycloartenone and cycloartenol and fructans; the first fractionation was performed on a polar chromatography column (silica gel; 230–400 mesh), and the second fractionation was performed with an apolar column chromatography (LiChroprep^®^ RP-18 silica gel; 40–63 µm); both silicas were purchased from Merck (Merck-Millipore, Billerical, MA, USA). Fractions from both columns were monitored by TLC (silica gel 60 F254 and silica gel 60 RP-18 F254S, respectively) purchased from Merck. The reagents used were H_2_SO_4_ 1%. Identification of the compound cantalasaponin was carried out by NMR spectroscopic analysis.

### 4.2. Plant Material

The leaves of *Agave tequilana* were collected in Yautepec, Morelos, Mexico (18°51′14.8″ N; 99°03′00.7″ W; Alt. 1186 m). A sample was deposited in the National Herbarium of Mexico (MEXU, for its acronym in Spanish) at the National Autonomous University of Mexico (UNAM), with registration number 1419699. The identification was made by Abisaí Josué García Mendoza, Curator of the National Collection of Agaves, UNAM.

### 4.3. Acetone Extracts Fractionation

Plant material (chopped and lyophilized leaves, 3500 g dry weight) was extracted with 15 L of acetone by two exhaustive macerations for 48 h each in light at room temperature. The solvent was eliminated by reduced-pressure distillation with a Heidolph Laborota 4000 rotary evaporator. The dry extract was analysed by TLC. The total yield was 1.07%. The acetone extract (9.5 g) was fractionated in an open column chromatograph (5 × 60 cm) previously packed with 30 g of silica gel normal phase (70–230 mesh) (Merck-Millipore, Billerical, MA, USA). For the mobile phase an *n-*hexane-acetone gradient system was employed, starting with 100% of *n*-hexane solvent with 5% polarity increments, finalizing with 100% of acetone. Aliquots of 200 mL were collected, yielding 86 fractions. The separation was monitored by TLC. The reagents used were ceric ammonium sulphate (CAS) at 1% in H_2_SO_4_ 2N. Fractions I, II and III were analysed by NMR spectroscopy. Fraction IIA (also called RIC1) was observed as a major compound in the acetone extract composition; therefore, it was decided to do a further fractionation to identify this compound. Thus RIC1 was fractionated on an open column (3 × 40 cm) previously packed with 10 g of LiChroprep^®^ RP-18 (40–63 µm) silica gel; a water-acetonitrile gradient system was employed for the mobile phase, starting with 100% of the most polar solvent with 10% polarity changes finalizing with 100% acetonitrile, aliquots of 20 mL were collected yielding 77 fractions which were grouped by chemical similarities into eight groups (RIC1/G1–8). The separation process was monitored by TLC. The reagents applied were ceric ammonium sulphate (CAS) at 1% in H_2_SO_4_ 2 N. The main fractions obtained from the R1C1 were analyzed by gas chromatography-mass spectrometry; these fractions were named F6, F9, F53 and F57. Fructans was obtained by a standard methodology [41].

### 4.4. Animals and Experimental Design

Balb/c female mice 8 weeks of age were acquired at Harlan Laboratories SA de CV (Mexico City, CDMX, Mexico). Maintenance of the animals, studies and development of the experiments were carried out accordingly to the Official Mexican Norm 062-ZOO-1999 (Technical Specifications for the Production, Care and Use of Laboratory Animals) and the international ethical guidelines for the care and use of laboratory animals. Authorization for development the experimental protocol was authorized by the local Health Research Committee IMSS (Registration R-2013-1702-59). Seven study groups were formed with *n* = 12 animals each. The animals were maintained at a temperature of 22 ± 3 °C, at 70 ± 5% humidity, light-dark cycle of 12 h each and with free access to water and food. In the bioassays, only the basal group received a single dose of physiological saline solution injected intraperitoneally (via ip) and did not receive any other treatment (Basal); the other groups were injected with TMPD to develop SLE; the damage group also called negative control, received oral administration of the vehicle without any other drugs (Negative); the positive control group was administered methotrexate (Mtx) at a dose of 3 mg/kg, and a second positive control group was administered with prednisone (Pdn) at a dose of 2.5 mg/kg; and three groups of *Agave tequilana* were used for the trials; the first group received acetone extract (ExA) at a dose of 50 mg/kg; the second group, received fructans (Fr) at a dose of 50 mg/kg; and the third received a mixture of acetone extract and fructans (MxAFr) at a dose of 100 mg/kg. The dosing schedule was designed based on the following considerations: traditional medicine recommends the consumption of 50 mL per dose of leaf juice, which implies approximately 3.5 g solids. For a human adult of 70 kg, this would represent a dose of around 50 mg/kg.

### 4.5. Induction of Systemic Lupus Erythematosus (SLE) Model by Administering Pristane

The methodology used for the induction of SLE was taken from that reported by Satoh and Reeves [24]; where a single administration of 0.5 mL of 2,6,10,14-tetramethylpentadecane (TMPD, also known as pristine) was injected via ip; according to this authors, pristane has the ability to trigger the production of autoantibodies characteristic of SLE and according to [25] the development of SLE by TMPD in Balb/c mice occurs seven months post-injection of the TMPD. Therefore, taking in to account the development of the disease that occurs in a period of seven months after the injection of a single dose of TMPD, Mtx, Pdn, ExA, Fr and MxAFr were administered during the first, third, fifth and seventh month after pristine was injected. Months two, four and six were rest periods, this was done since pharmacological treatments should have rest periods during their use. Therefore, the assay was carried out following the progress of the disease, recording the levels of protein in urine (proteinuria) and the evolution of arthritis by measure the size of the mice joints, they were measured at the following period: zero, two, four and seven months. The urine samples for proteinuria evaluation were obtained by means of metabolic cages and were analyzed with quantitative and semi-quantitative methods. Arthritis development was assessed quantitatively by measuring the size of the joints using a digital 150 mm/6 inch Vernier caliper (World Precision Instruments Inc., Sarasota, FL, USA).

Seven months after the injection of TMPD, the animals of all the groups were anaesthetized with sodium pentobarbital via ip, at a dose of 55 mg/kg. With the sedated animals, blood samples were collected, 1.5 mL were obtained from the retro-orbital venous sinus; the blood was centrifuged to separate the red cells from the serum. The serum was stored at −70 °C for later use for the evaluation of antinuclear autoantibodies with ELISA method. After blood recollection, the next step was to obtain the kidneys which were stored at −70 °C until homogenization for the determination of proinflammatory (IL-1β, IL-6, TNF-α, IFN-γ) and anti-inflammatory (IL-10) cytokines by an ELISA method.

### 4.6. Obtaining of Urine Samples, Blood Serum, and Kidney Homogenates

To obtain the urine samples, each group with 12 mice each were divided into three metabolic cages (four mice per metabolic cage). The study animals were maintained in the metabolic cages for a period of 24 h, with free access to food and water. At the end of this period, animals were removed from the metabolic cages, and the urine that was collected was decanted into a glass tube to be centrifuged at 2000 rpm for 7 min. After centrifugation, 0.5 mL of supernatant was used for the semi quantitative analysis of urine protein, the evaluation was done by dipping a strip of Combur10Test and for the quantitative analysis, and 5 µL of the urine supernatant was used for the spectrophotometric procedure with 250 µL of Bradford reagent in a 96 well plate.

To obtain the blood serum sample, was made by introducing a heparinized capillary into the mouse retro-orbital venous sinus where 1.5 mL of blood sample was obtained, which was kept in ice until processing; then, red cell plasma was separated by centrifugation at 5000 rpm for 10 min, the resulting serum was stored at −70 °C until the analysis of antinuclear autoantibodies. 

For kidney homogenization, after the blood samples were taken, both kidneys of each mouse were obtained and treated by the following protocol: (a) the tissue was partially ground; (b) the homogenate was placed into a glass vial where, for each gram of tissue the following solution was added, 4 mL of a prepared solution of phosphate buffered saline (PBS; pH 7.4) with phenylmethyl-sulfonyl fluoride (PMSF) at 0.01% dissolved in isopropyl alcohol (VWR International) (ratio 100:1 PBS:PMFS); (c) the above mixture was homogenized for 15 s with an Ultra Turrax homogenizer T-10, the homogenization was repeated five consecutive times with 30 s intervals between homogenizations; (d) finally, the samples were centrifuged at 12,000 rpm for 5 min and 300 µL aliquots of the supernatant were obtained and stored at −70 °C; each aliquoted served to quantify a different cytokine.

### 4.7. Quantification of Antinuclear Antibody Anti-ssDNA and Anti-dsDNA by ELISA

Quantification of antinuclear antibodies (anti-DNA and anti-dsDNA) was performed by using the mouse ELISA kit (MyBioSource.com). Following the manufacturer’s instructions; briefly the reagents and samples were placed at room temperature 20 min before the test. In a 96-well plate are placed 100 µL of samples or standards, the plates sealed with adhesive tape. The plate was incubated at 37 °C for 90 min. At the end of the period the plate was washed three times with washing solution. Then 100 µL of the biotinylated mouse antibody was added and the plate was again sealed with adhesive tape and incubated at 37 °C for 30 min., at the end of the incubation, 5 washes were performed. In the next step 100 µL of the color developer was added and the plate was incubated this time at 30 °C in the dark for 30 min. The reaction was stopped when the colour gradients for the standard appeared. The plates were mixed and read in the Stat Fax 2100 spectrophotometer (Awareness Technologies, Palm City, FL, USA) at 450 nm within 10 min after the addition of the last reagent.

### 4.8. Quantification of Cytokines by ELISA

The quantification of different cytokines (IL-1, IL-6, TNF-, IFN- and IL-10) was carried out with an ELISA method using a OptEIATM ELISA kit purchased from BD Biosciences, following the manufacturer’s instructions. Briefly, using 96-well plates 100 µL/well of the capture antibody was added; the plates were incubated for 12 h at 4 °C. Once this time had passed, the plates were washed with PBS buffer (0.05% of Tween-20, 300 µL/well × 3 times), followed by the addition of 100 µL/well of PBS with fetal bovine serum (FBS) at 10%, pH 7.0, for a period of 1 h at room temperature. The contents were discarded and the plates were washed three times with PBS buffer. Subsequently, 100 L of either concentration standards or samples were added and then the plate was incubated at room temperature for 2 h. The contents of the plates were discarded and the plates were washed five times with PBS buffer. Depending on the type of cytokines variations were made to each procedure, for IL-6, TNF-, IL-10 and IFN-, 100 µL/well of antibody detection plus streptavidin-horseradish peroxidase (HRP) enzyme was added and incubated for 1 h, following with seven washings with PBS buffer for IL-6, TNF- and IL-10, and 10 washings for IFN-. For IL-1 analysis 100 µL/well of antibody detection was added and incubated for 1 h and washed five times with PBS buffer, followed by the addition of streptavidin-HRP enzyme (100 µL/well), which was incubated at room temperature and washed seven times with PBS buffer. Finally, 100 µL of *o*-phenylendiamine (OPD) substrate was added to all plate. The plates were incubated for 30 min at room temperature in total darkness conditions, and then a stop solution (2 N H_2_SO_4_) was added. Reading of the plates were carried out in an Awareness Technologies Stat Fax 2100 spectrophotometer at a 450 nm wavelength at 37 °C, within 10 min after the addition of the stop reagent.

### 4.9. Statistical Analysis

The results were expressed as the average ± the Standard error of the mean (SEM), and the data was analysed using an Analysis of variance (ANOVA) two-way and the post hoc Bonferroni test (* *p* < 0.05). The employed statistical software package was IBM SPSS Statistics V. 22 (International Business Machines Corporation, Armonk, NY, USA).

## 5. Conclusions

In the present work we demonstrated the immunomodulatory effect of *Agave tequilana* on the chronic inflammation associated with SLE caused by the administration of TMPD to female Balb/c mice.

## Figures and Tables

**Table 1:** Phytochemical analysis of the acetone extract composition. The reported relative concentration corresponds to 1 g of extract.

Compounds	Relative Concentration	Quantity
Cycloartenol	0.0000889%	0.019 mg
Stigmasta-3,5-dien-7-one	0.0003%	0.0285 mg
Octadecadienoic acid 2,3-dihydroxypropyl ester	0.0012%	0.114 mg
3,7,11,15-Tetramethyl-2-hexadecen-1-ol	0.0017%	0.1615 mg
Β-Sitosterol glucoside	0.0021%	0.1995 mg
*n*-Hexadecanoic acid	0.0032%	0.304 mg
Octadecanoic acid	0.0052%	0.494 mg

## Supplementary Materials

The following are available online. Table S1: Results of the identification of the compounds by gas chromatography-mass spectrometry. Fraction 6 (F6). Table S2: Results of the identification of the compounds by gas chromatography-mass spectrometry. Fraction 9 (F9). Table S3: Results of the identification of the compounds by gas chromatography-mass spectrometry. Fraction 53 (F53). Table S4: Results of the identification of the compounds by gas chromatography-mass spectrometry. Fraction 57 (F57).
