# Supplementary material for: Immunomodulatory Effect of Agave tequilana Evaluated on an Autoimmunity Like-SLE Model Induced in Balb/c Mice with Pristane
Source: Molecules. 2017 May 25;22(6):848. doi: 10.3390/molecules22060848 (PMC6152617; doi:10.3390/molecules22060848)
Supplement: Supplementary file 1 [file molecules-22-00848-s001.pdf]

molecules

Table S1 . Results of the identification of the compounds by gas chromatography-mass spectrometry. Fraction 6 (F6)

Acq on: 18 Aug 2016 14:46. Sample: F6 Spitless 70°C-1min, 5°C/min-250°C-1min,10°C/min-285°C-20min

peak #	R.T. min	first scan	max scan	last scan	PK TY	peak height	corr. area	corr. % max.	% of Total	Compound
1	17.324	1702	1712	1721	BB2	458954	11486235	3.04%	1.571%	
2	25.115	2887	2898	2908	BV	1676696	39819245	10.54%	5.446%	Phytol (M)3,7,11,15-Tetramethyl-2-hexadecen-1-ol
3	25.614	2966	2974	2984	M	434926	10343307	2.74%	1.415%	Phytol
4	25.975	3020	3029	3038	M2	612451	14932192	3.95%	2.042%	Phytol
5	33.608	4182	4191	4204	BB	661849	16561603	4.38%	2.265%	Tricosane
6	36.826	4673	4681	4696	VB	835634	22470938	5.95%	3.073%	(R)Pentacosane
7	39.874	5139	5145	5156	M	1062929	24270709	6.42%	3.320%	(R)Heptacosane
8	40.761	5272	5280	5293	M	806011	17930458	4.75%	2.452%	Phytol
9	41.011	5312	5318	5321	M	529393	12888272	3.41%	1.763%	(M)Cyclooctacosane
10	41.050	5321	5324	5335	M	604744	12842560	3.40%	1.756%	(M)Octacosane
11	42.108	5474	5485	5500	M	1061467	30029310	7.95%	4.107%	(M)Nonacosane
12	43.231	5642	5656	5673	M3	615916	25917591	6.86%	3.545%	(M)1-Dotriacontanol
13	44.617	5850	5867	5878	M	942314	34000389	9.00%	4.650%	(M)Hentriacontane
14	44.768	5879	5890	5909	M4	1546668	54924996	14.54%	7.512%	Phytol
15	50.745	6775	6800	6826	BB	417987	24936726	6.60%	3.411%	(M)Stigmasta-3,5-dien-7-one
16	55.396	7440	7508	7558	BB 4	3172707	377800732	100.00%	51.672%	Phytol

Sum of corrected areas: 731155261

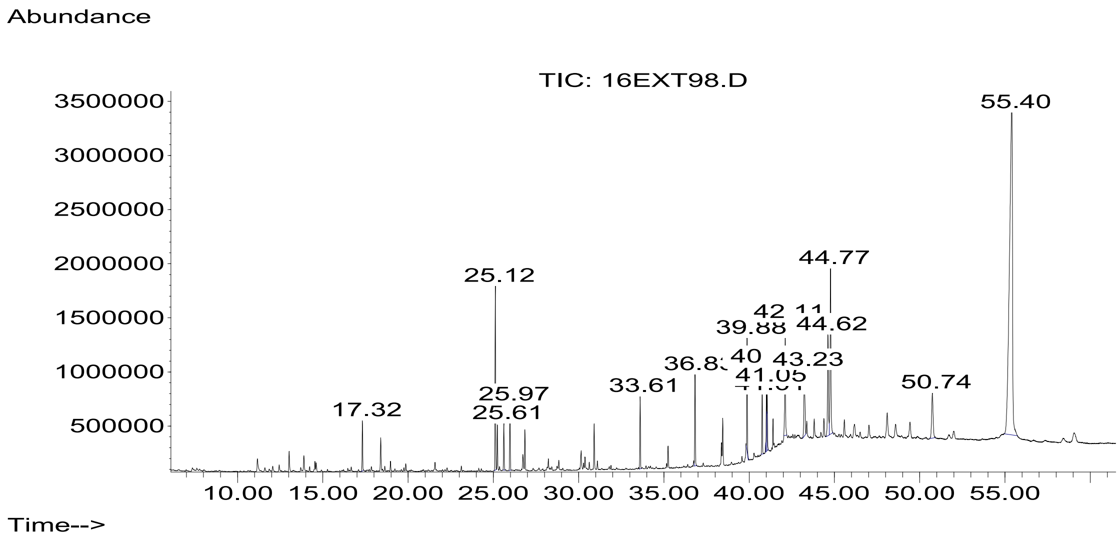

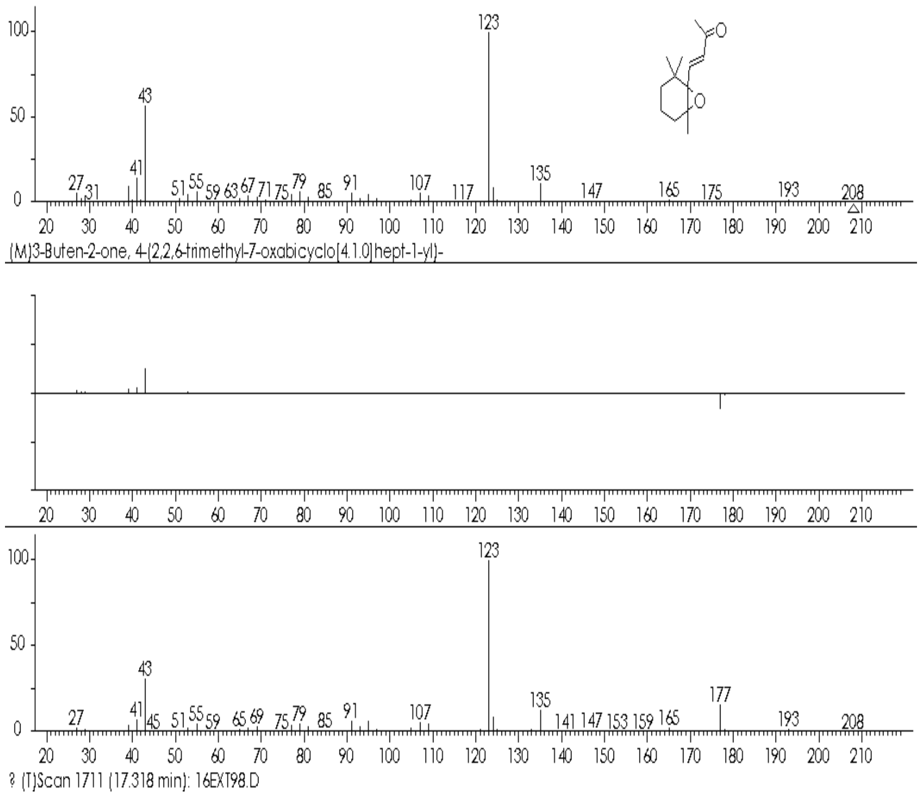
(M)3-Buten-2-one, 4-(2,2,6-trimethyl-7-oxabicyclo[4.1.0]hept-1-yl)-
? (T)Scan 1711 (17.318 min): 16EXT98.D

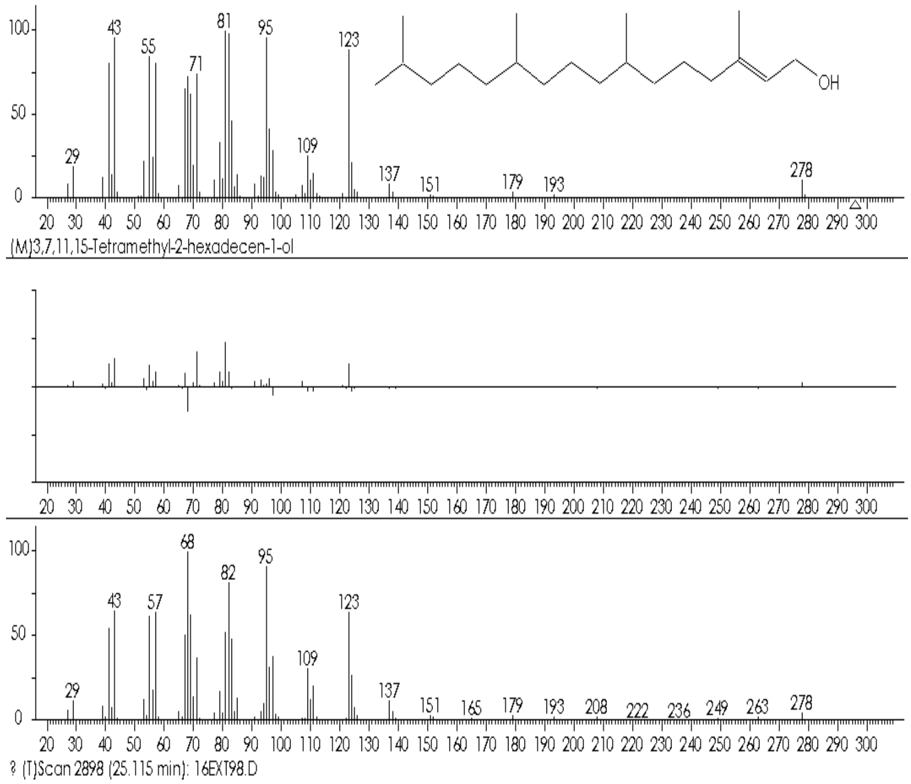
OH
(M)3,7,11,15-Tetramethyl-2-hexadecen-1-ol
? (T)Scan 2898 (25.115 min): 16EXT98.D

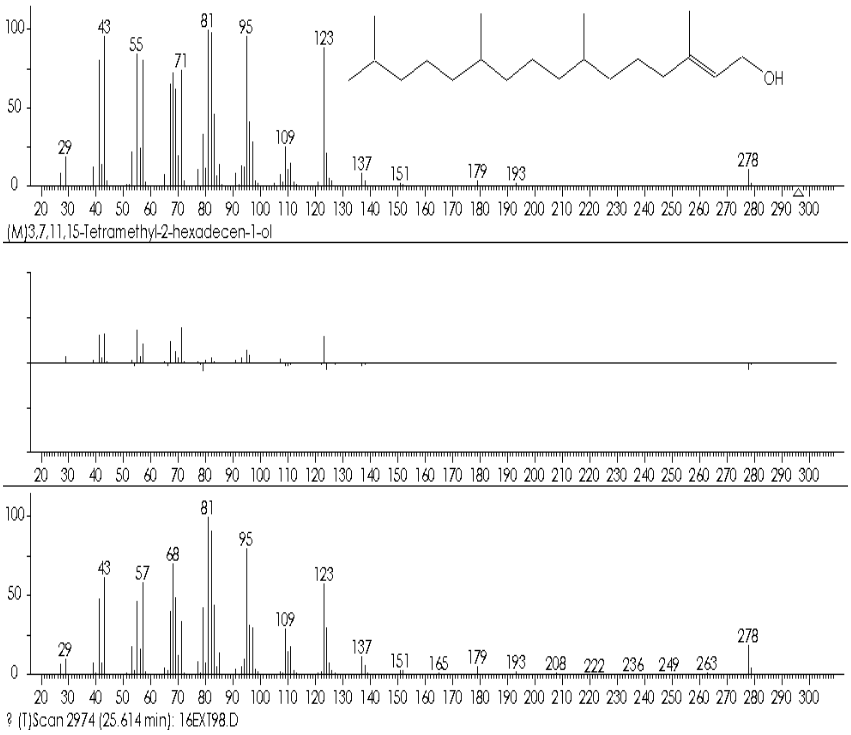
(M)3,7,11,15-Tetramethyl-2-hexadecen-1-ol
OH
? (T)Scan 2974 (25.614 min): 16EXT98.D

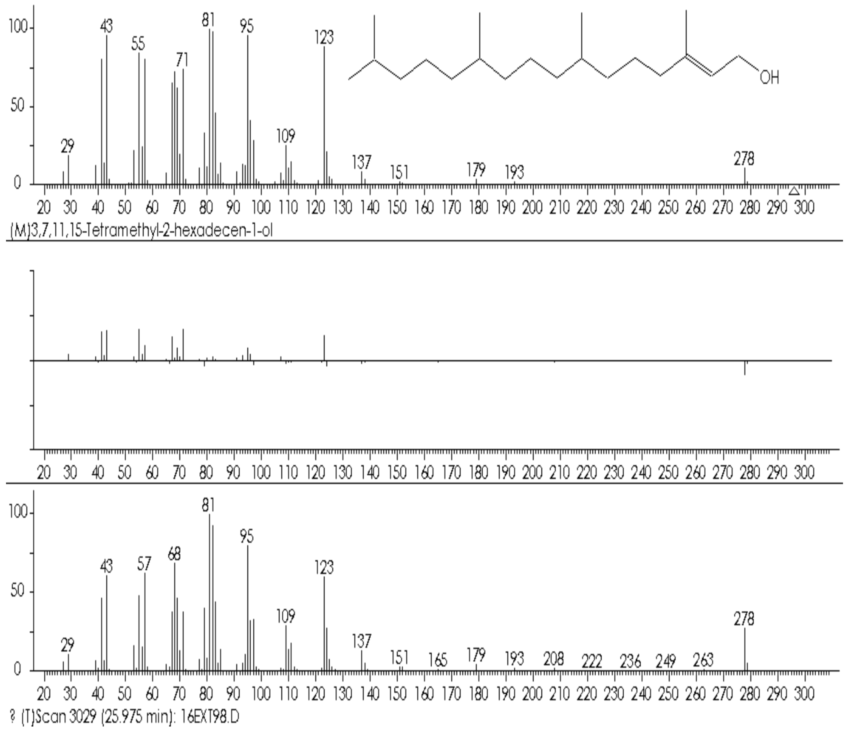
(M)3,7,11,15-Tetramethyl-2-hexadecen-1-ol
OH
? (T)Scan 3029 (25.975 min): 16EXT98.D

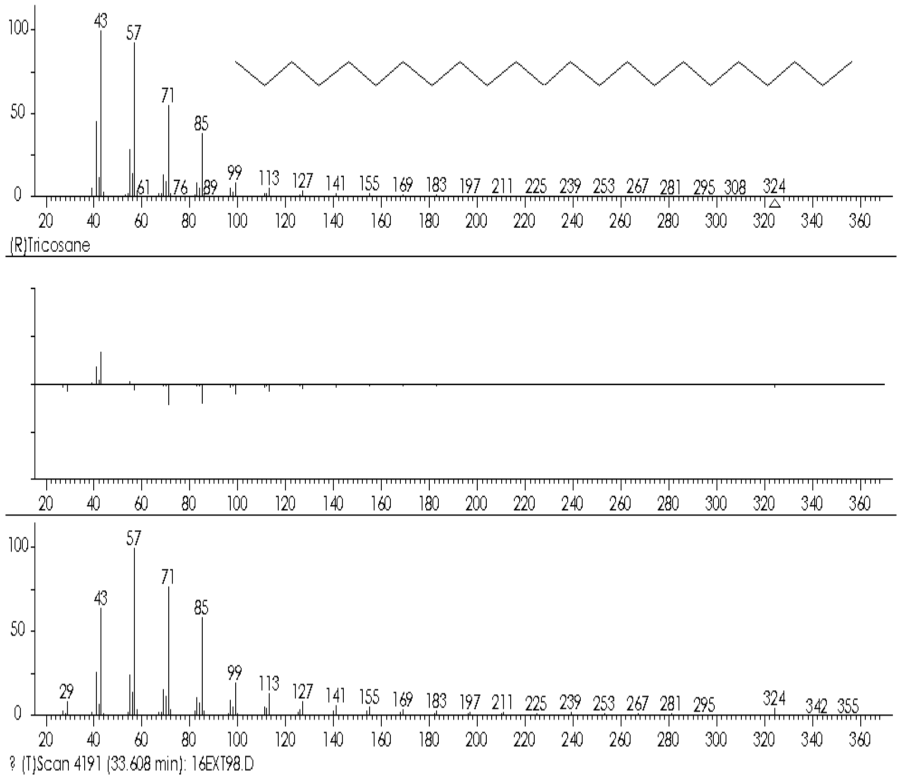
(R)Tricosane
? (T)Scan 4191 (33.608 min): 16EXT98.D

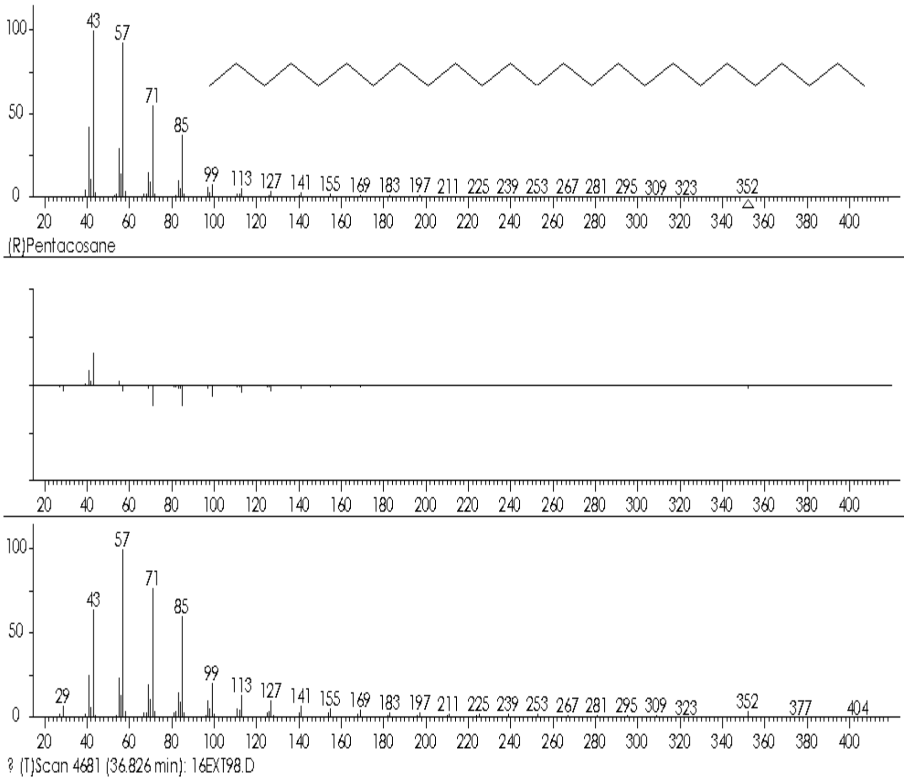
(R)Pentacosane
? (T)Scan 4681 (36.826 min): 16EXT98.D

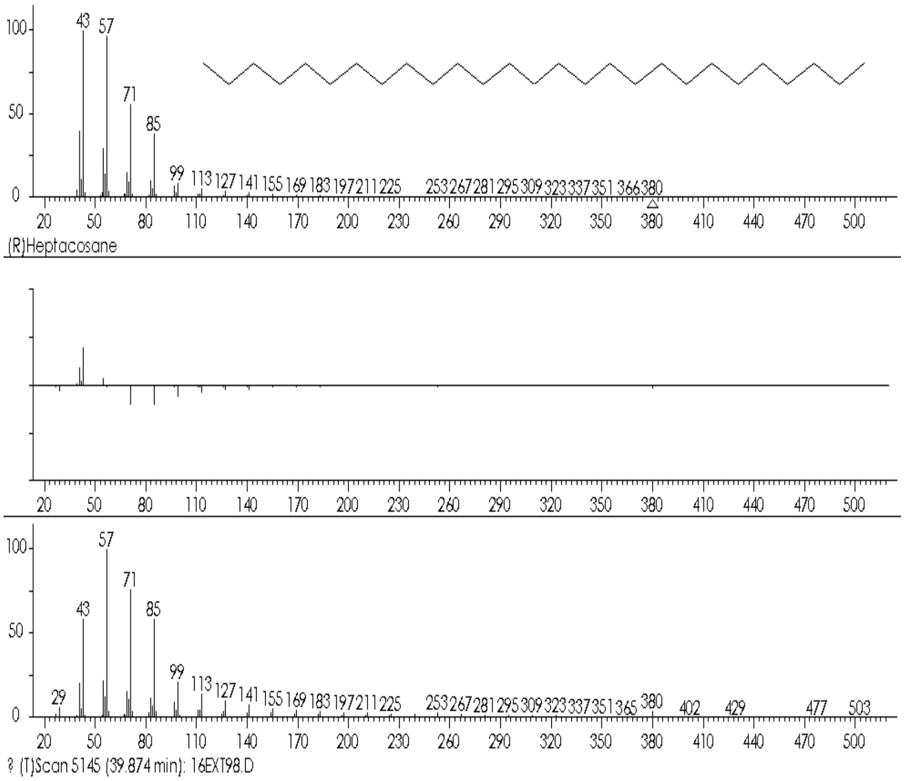
(R)Heptacosane
? (T)Scan 5145 (39.874 min): 16EXT98.D

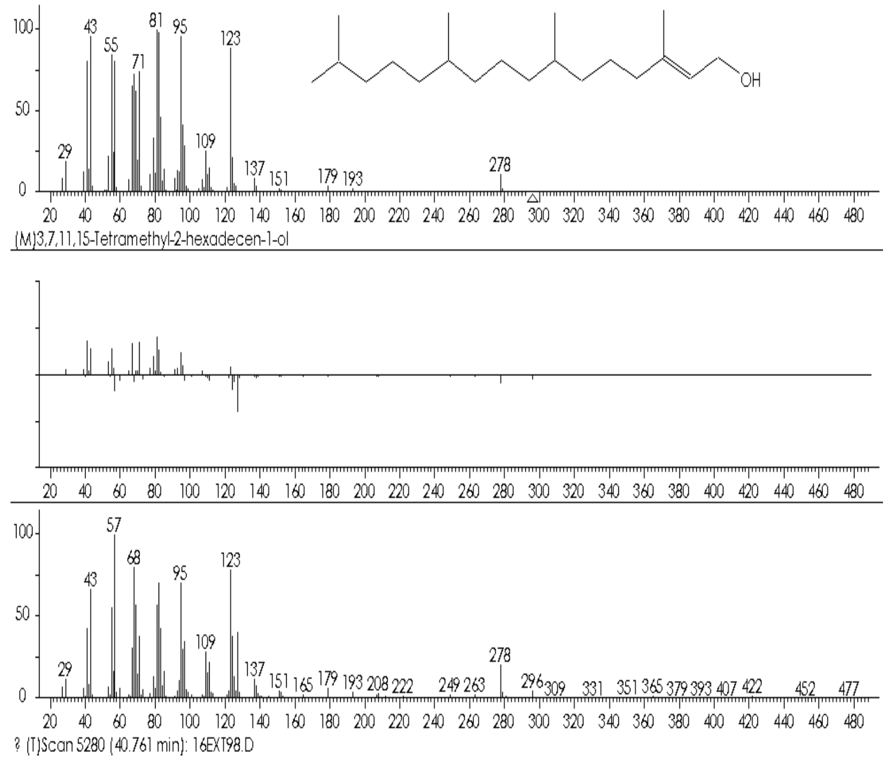
OH
(M)3,7,11,15-Tetramethyl-2-hexadecen-1-ol
? (T)Scan 5280 (40.761 min): 16EXT98.D

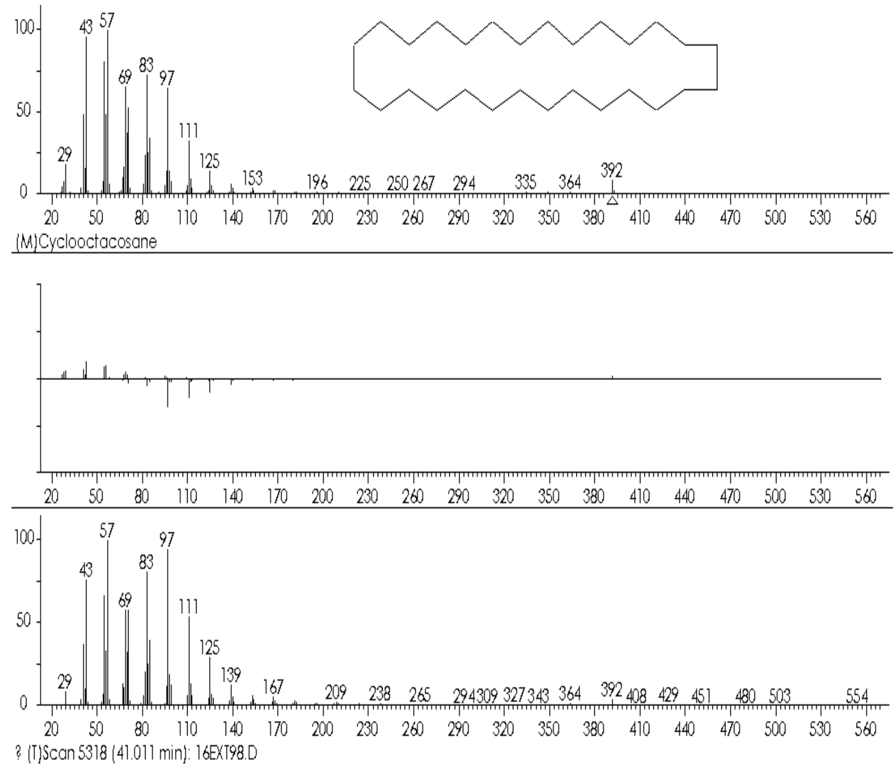
43
57
83
69
97
111
29
125
153
196
225
250 267
294
335
364
392
(M)Cyclooctacosane
43
57
97
83
69
111
125
139
167
29
209
238
265
294309
327343
364
392
408
429
451
480
503
554
? (T)Scan 5318 (41.011 min): 16EXT98.D

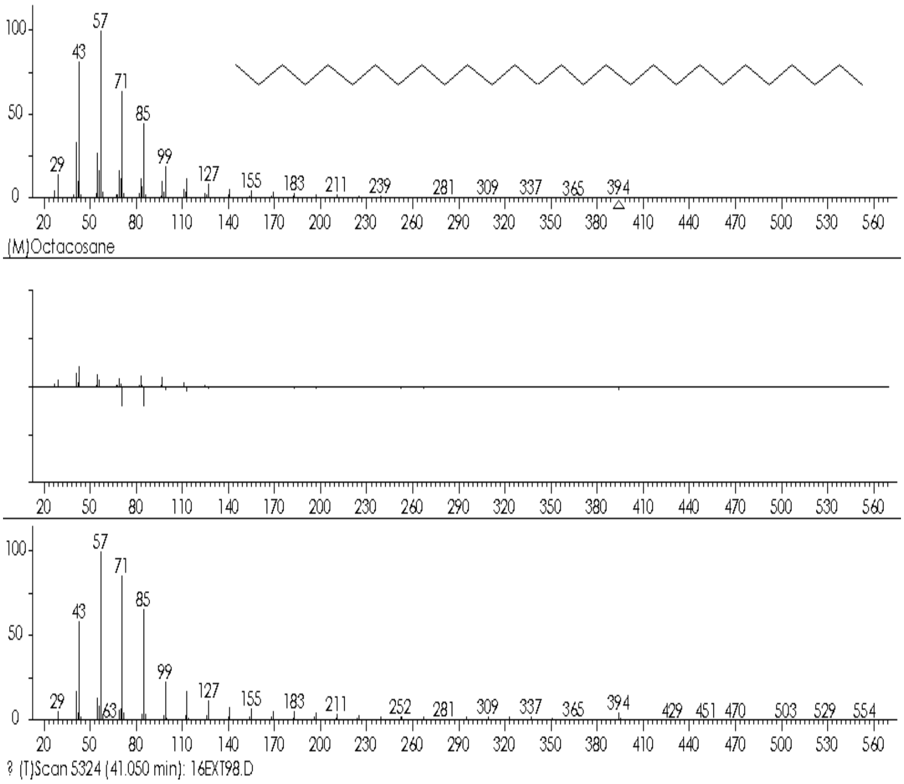
57
43
71
85
99
29
127
155
183
211
239
281
309
337
365
394
(M)Octacosane
57
71
85
43
99
127
155
183
211
252
281
309
337
365
394
429
451
470
503
529
554
29
63
? (T)Scan 5324 (41.050 min): 16EXT98.D

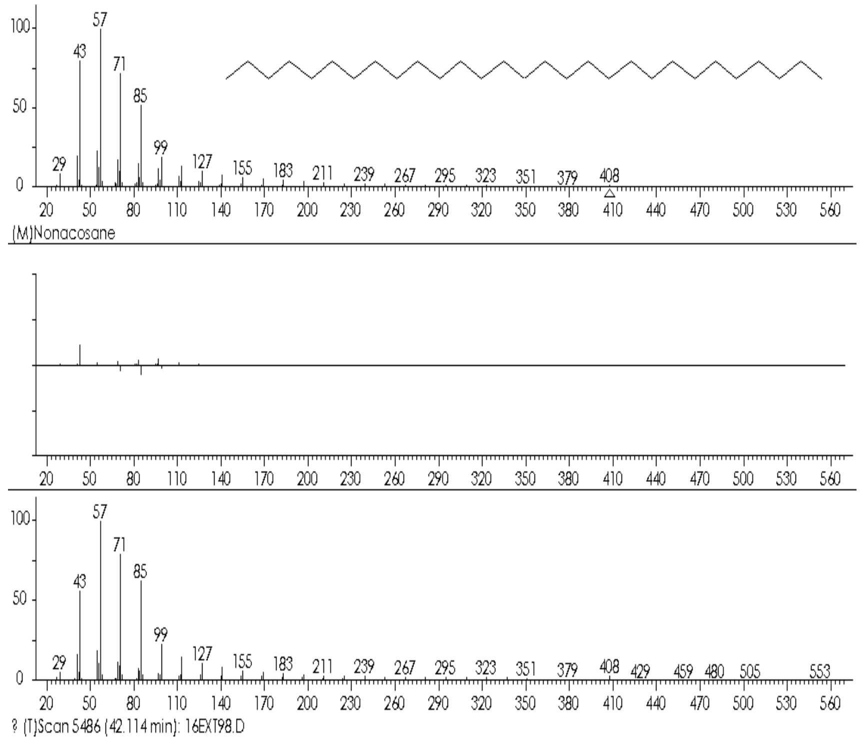
(M)Nonacosane
? (T)Scan 5486 (42.114 min): 16EXT98.D

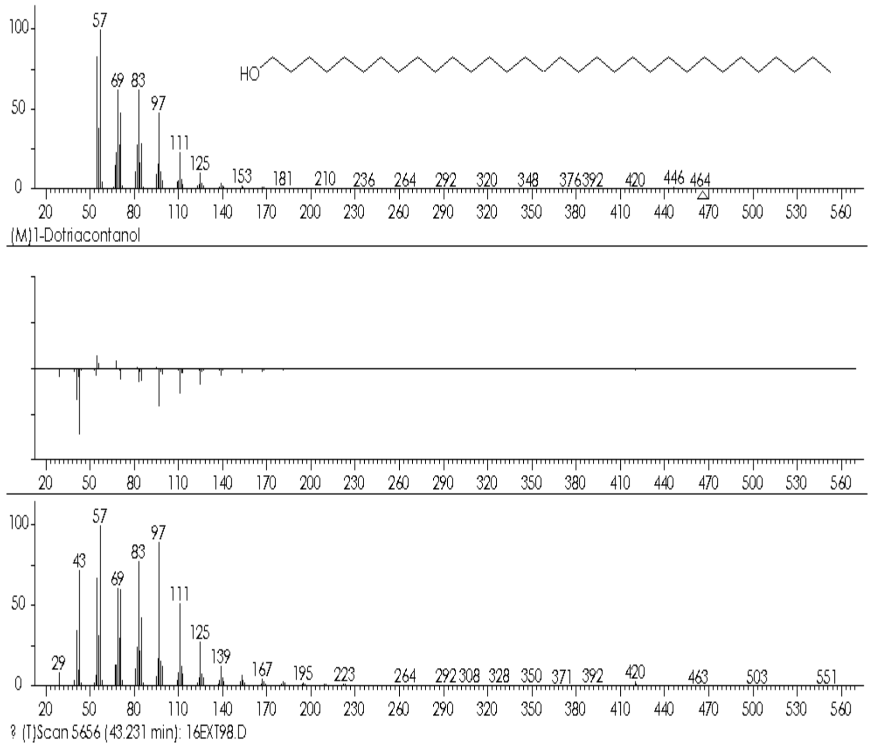
HO
(M)1-Dotriacontanol
? (T)Scan 5656 (43.231 min): 16EXT98.D

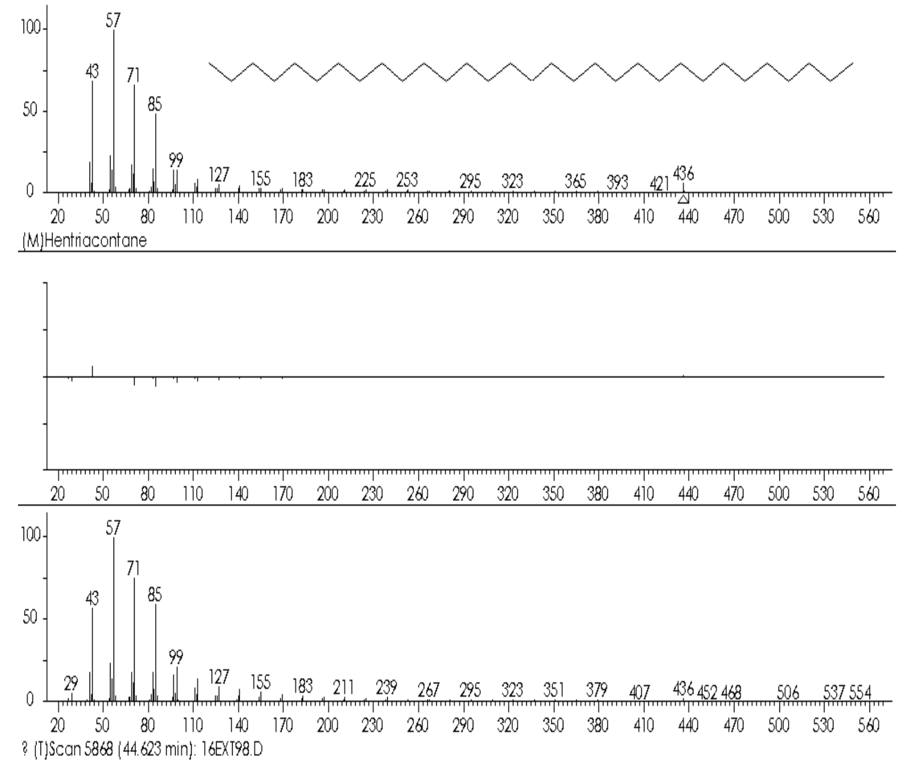
(M)Hentriacontane
? (T)Scan 5868 (44.623 min): 16EXT98.D

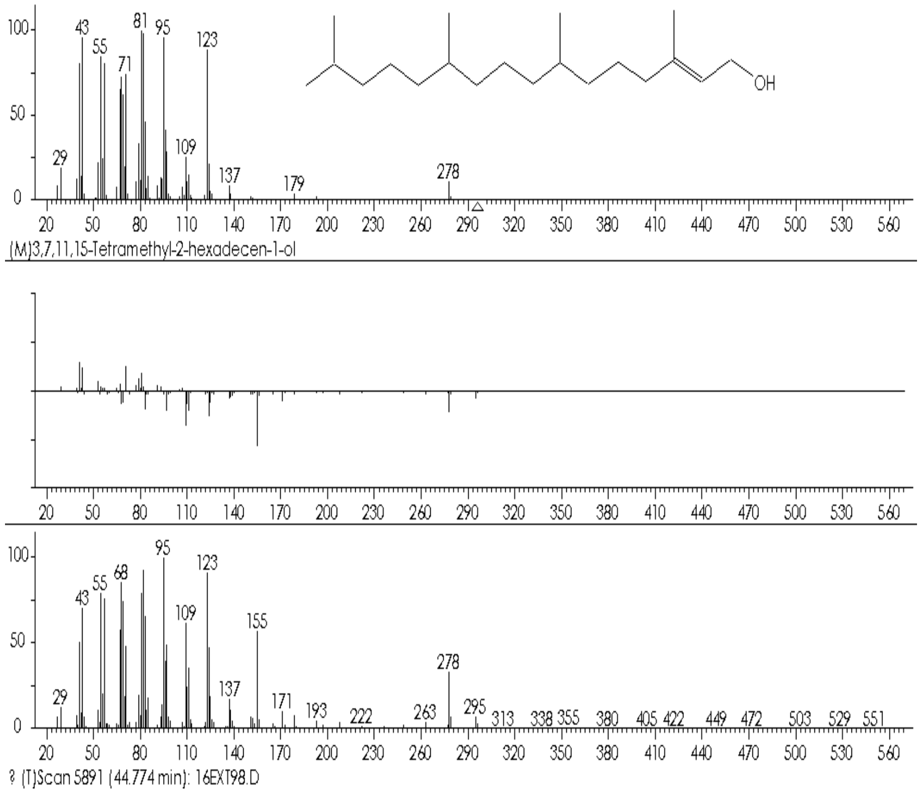
OH
(M)3,7,11,15-Tetramethyl-2-hexadecen-1-ol
? (T)Scan 5891 (44.774 min): 16EXT98.D

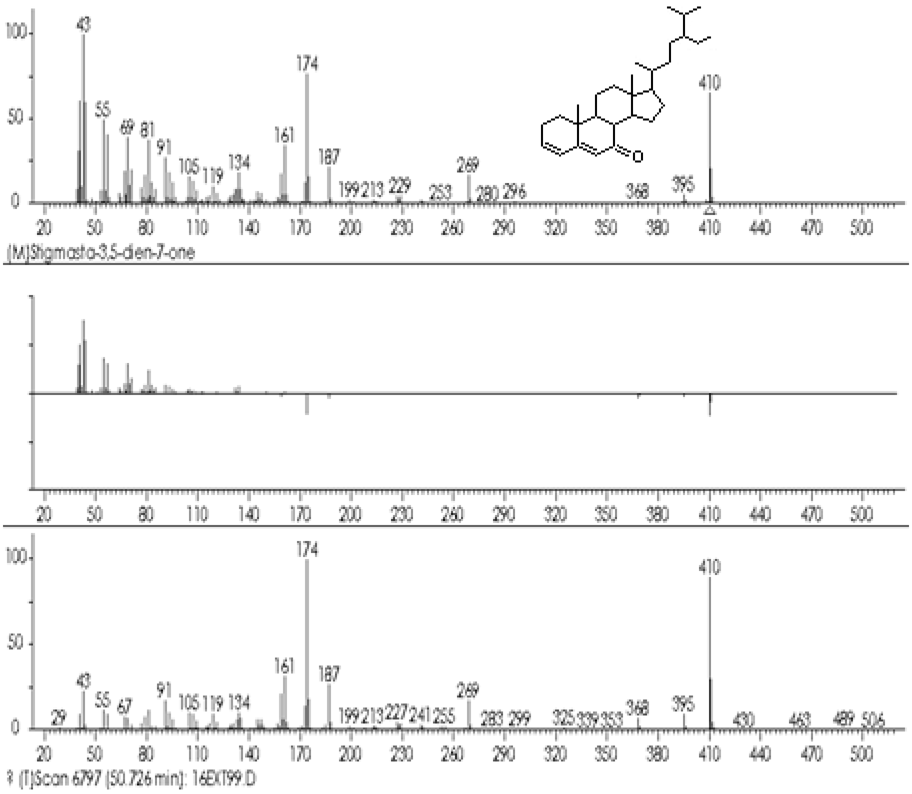
(M)Stigmasta-3,5-dien-7-one
O
? (T)Scan 6797 (50.726 min): 16EXT99.D

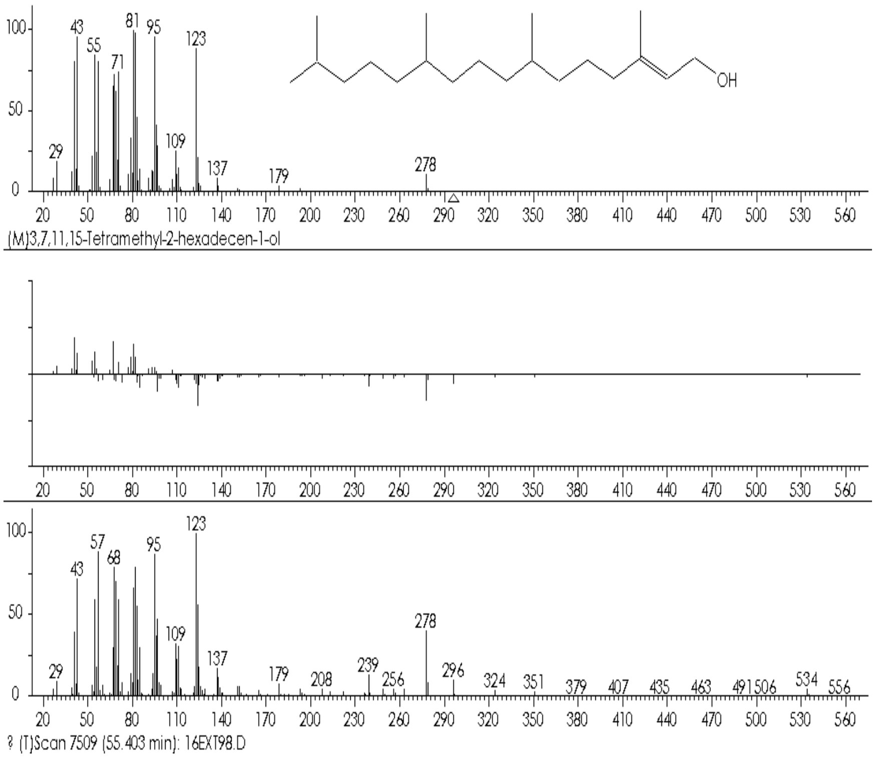
OH
(M)3,7,11,15-Tetramethyl-2-hexadecen-1-ol
? (T)Scan 7509 (55.403 min): 16EXT98.D

Table 2S . Results of the identification of the compounds by gas chromatography-mass spectrometry.

Fraction 9 (F9)

Acq On: 18 Aug 2016 17:08 Sample: F9 Spitless 70°C-1min, 5°C/min-250°C-1min,10°C/min-285°C-20min

peak #	R.T. min	first scan	max scan	last scan	PK TY	peak height	corr. area	corr. % max.	% of Total	Compound
1	26.849	3154	3162	3173	VB	846626	20920338	1.20%	0.629%	Hexadecanoic acid, methyl ester
2	28.051	3256	3345	3451	BB	6264846	877772570	50.45%	26.411%	(R) n-Hexadecanoic acid
3	30.054	3636	3650	3658	BV	1082233	26593202	1.53%	0.800%	(M) 9,12-Octadecadienoic acid (Z,Z)-methyl ester
4	30.166	3658	3667	3680	PB 2	1009442	27254592	1.57%	0.820%	(M) 8-Octadecenoic acid, methylester
5	30.474	3691	3714	3724	M	9151386	413919542	23.79%	12.454%	(R) Phytol
6	31.387	3757	3853	3935	M	8349820	1739770393	100.00%	52.347%	(R) 9,12-Octadecadienoic acid (Z,Z)
7	47.047	6218	6237	6245	M4	1226908	61532671	3.54%	1.851%	(M) 1,30-Triacontanediol
8	48.597	6449	6473	6498	BB4	546881	31432522	1.81%	0.946%	(R) τ–Sitosterol
9	49.018	6514	6537	6551	BV3	560998	29554610	1.70%	0.889%	(M) Cycloartenol
10	49.241	6551	6571	6600	VB7	623394	39758319	2.29%	1.196%	(M) Pollinastanol
16	50.259	6701	6726	6760	BB4	889718	55041731	3.16%	1.656%	(M) 9,19-Cyclolanost-24-en-3-ol, (3β) -

Sum of corrected areas: 3323550491

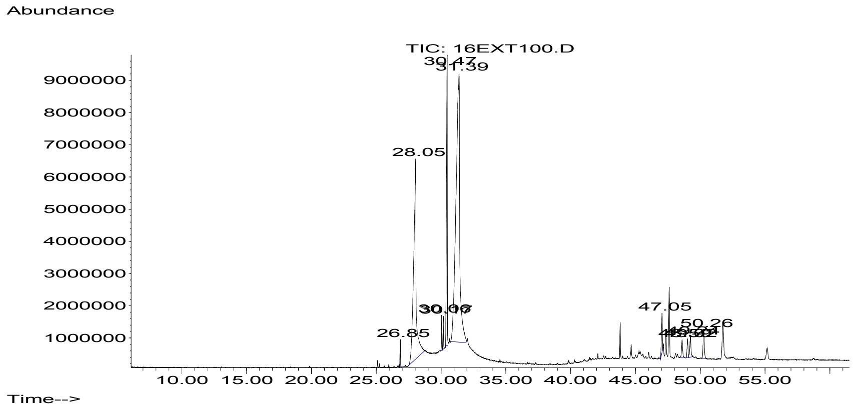

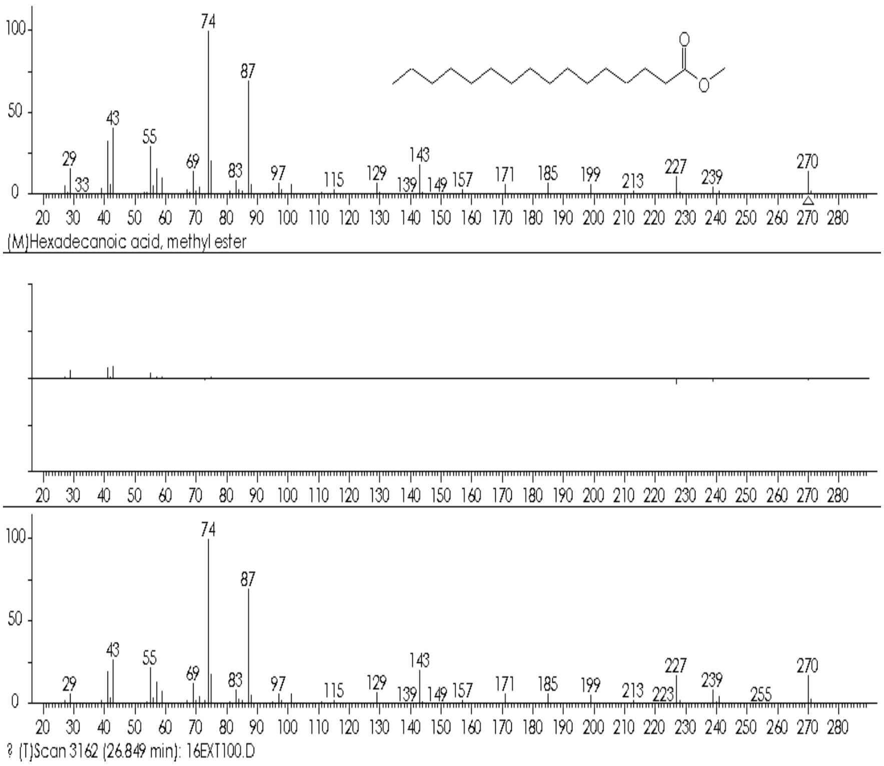
(M)Hexadecanoic acid, methyl ester
? (T)Scan 3162 (26.849 min): 16EXT100.D

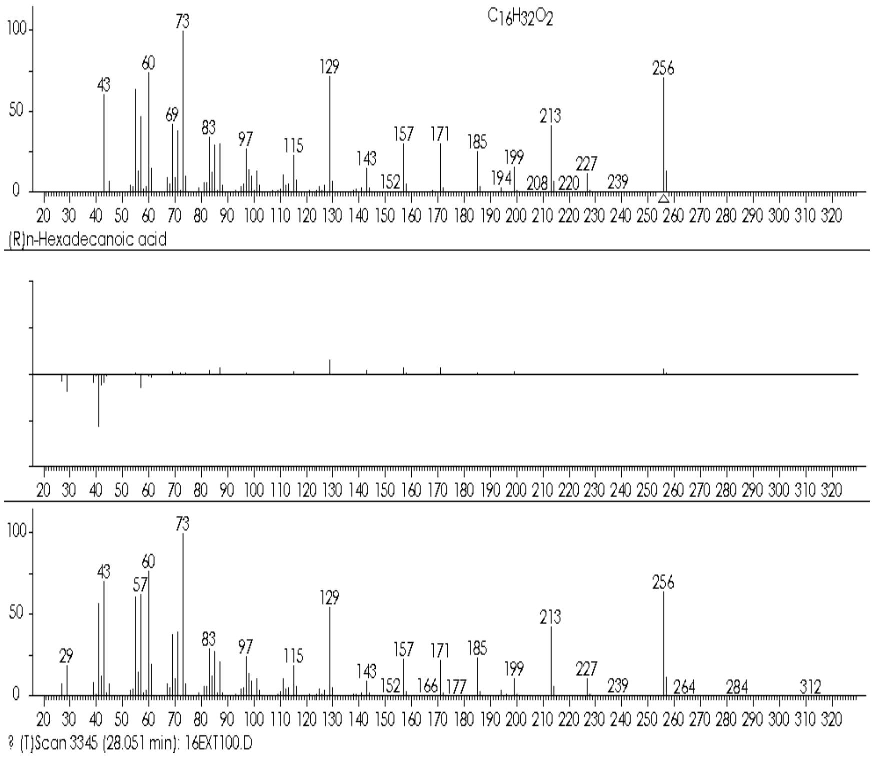
C16H32O2
(R)n-Hexadecanoic acid
? (T)Scan 3345 (28.051 min): 16EXT100.D

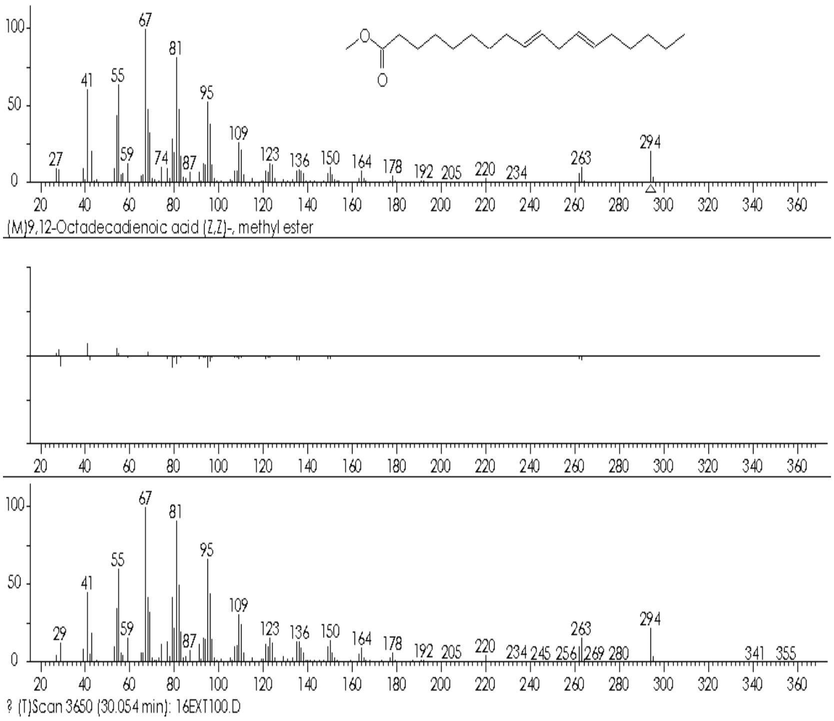
(M)9,12-Octadecadienoic acid (Z,Z)-, methyl ester
? (T)Scan 3650 (30.054 min): 16EXT100.D

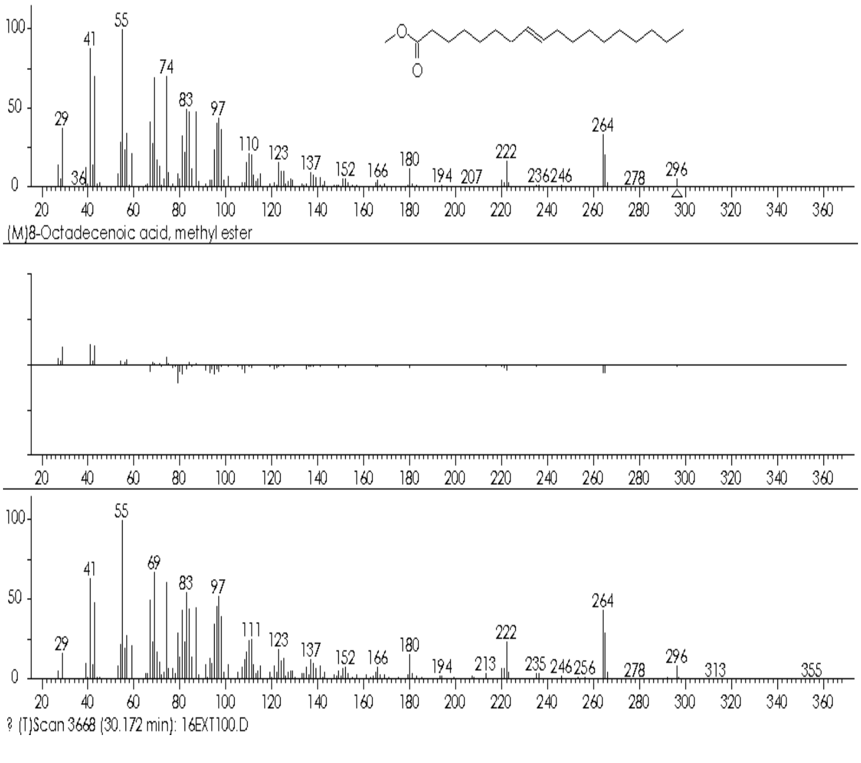
(M)8-Octadecenoic acid, methyl ester
? (T)Scan 3668 (30.172 min): 16EXT100.D

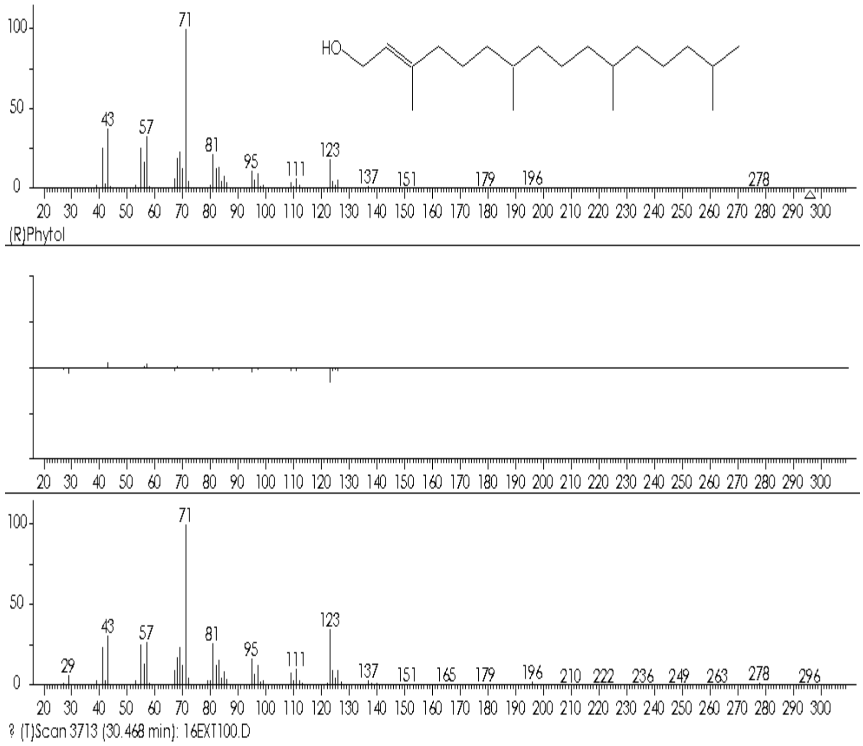
100
50
0
71
43
57
81
95
111
123
137
151
179
196
278
HO
20 30 40 50 60 70 80 90 100 110 120 130 140 150 160 170 180 190 200 210 220 230 240 250 260 270 280 290 300
(R)Phytol
29
165
210
222
236
249
263
296
? (T)Scan 3713 (30.468 min): 16EXT100.D

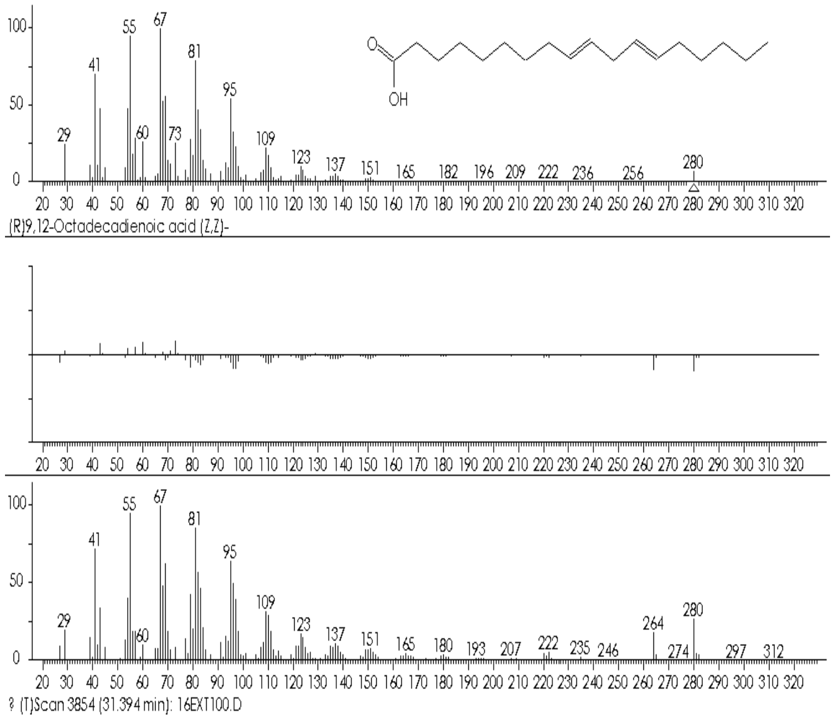
100
50
0
55
67
41
81
95
29
60
73
109
123
137
151
165
182
196
209
222
236
256
280
O
OH
20 30 40 50 60 70 80 90 100 110 120 130 140 150 160 170 180 190 200 210 220 230 240 250 260 270 280 290 300 310 320
(R)9,12-Octadecadienoic acid (Z,Z)-
180
193
207
235
246
264
274
297
312
? (T)Scan 3854 (31.394 min): 16EXT100.D

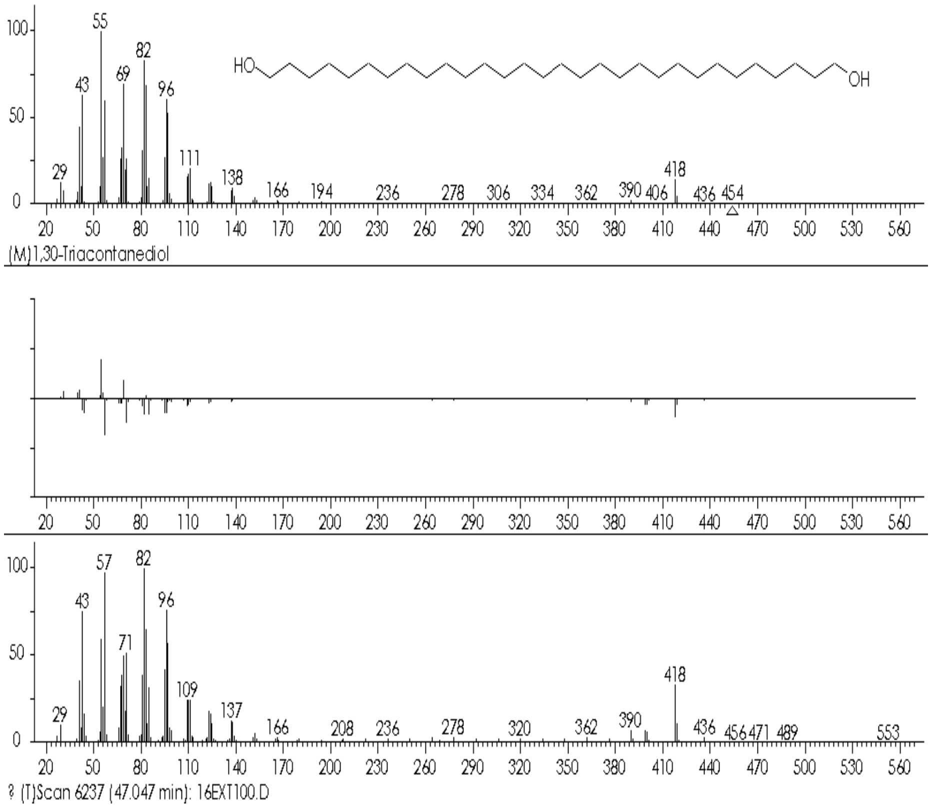
HO
OH
(M)1,30-Triacontanediol
? (T)Scan 6237 (47.047 min): 16EXT100.D

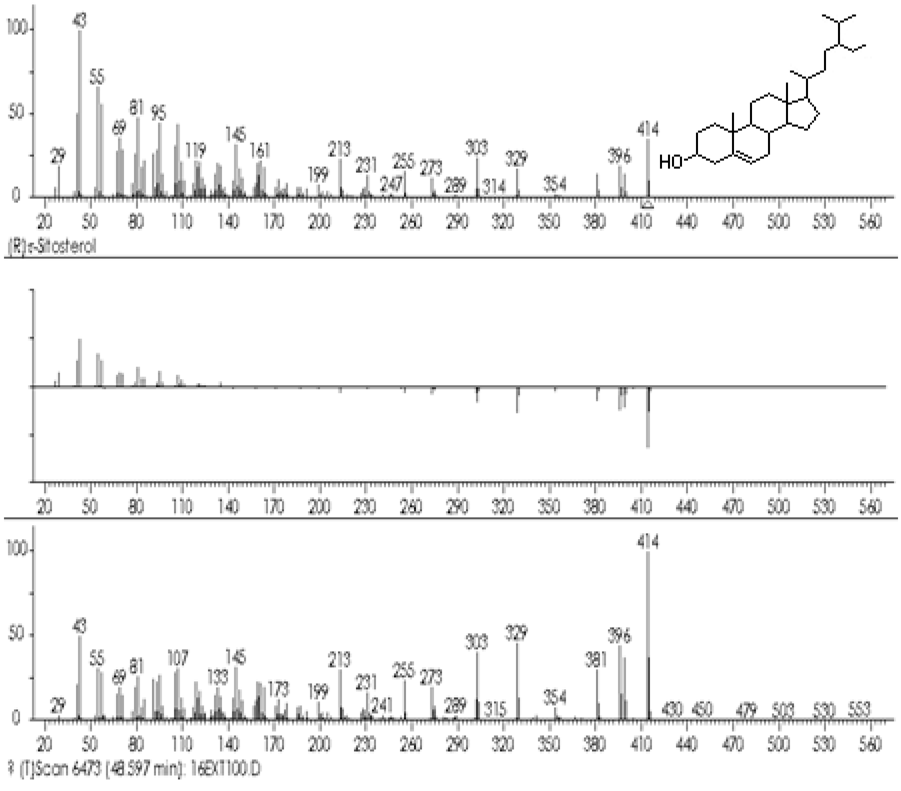
HO
(R)ε-Sitosterol
? (T)Scan 6473 (48.597 min): 16EXT100.D

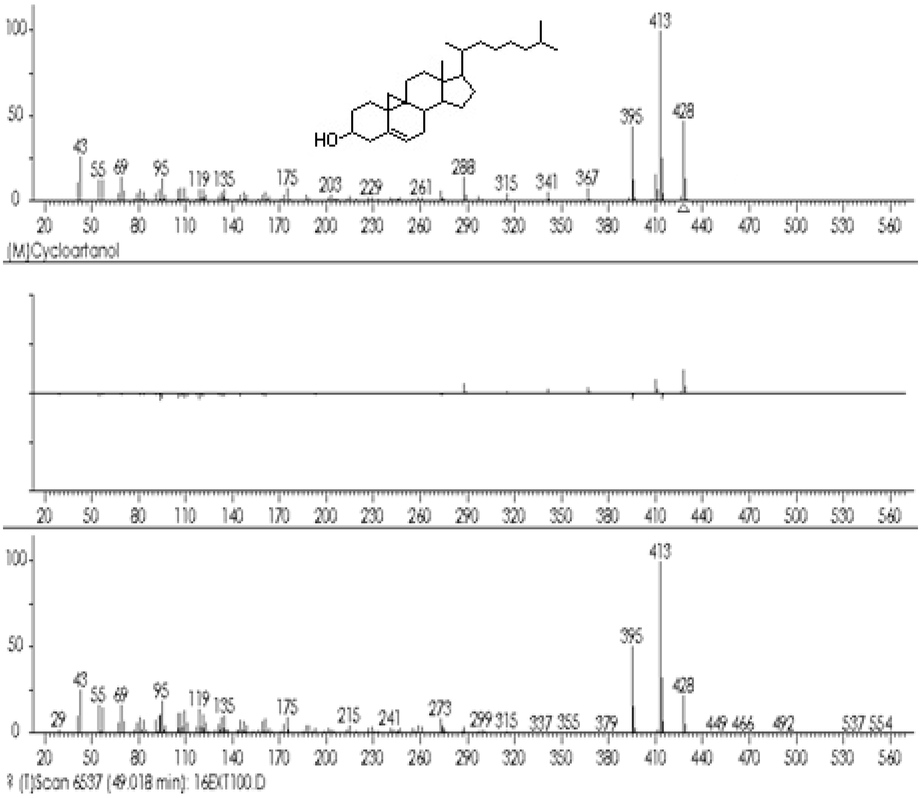
HO
(M)Cycloartanol
(T)Scan 6537 (49.018 min): 16EXT100.D

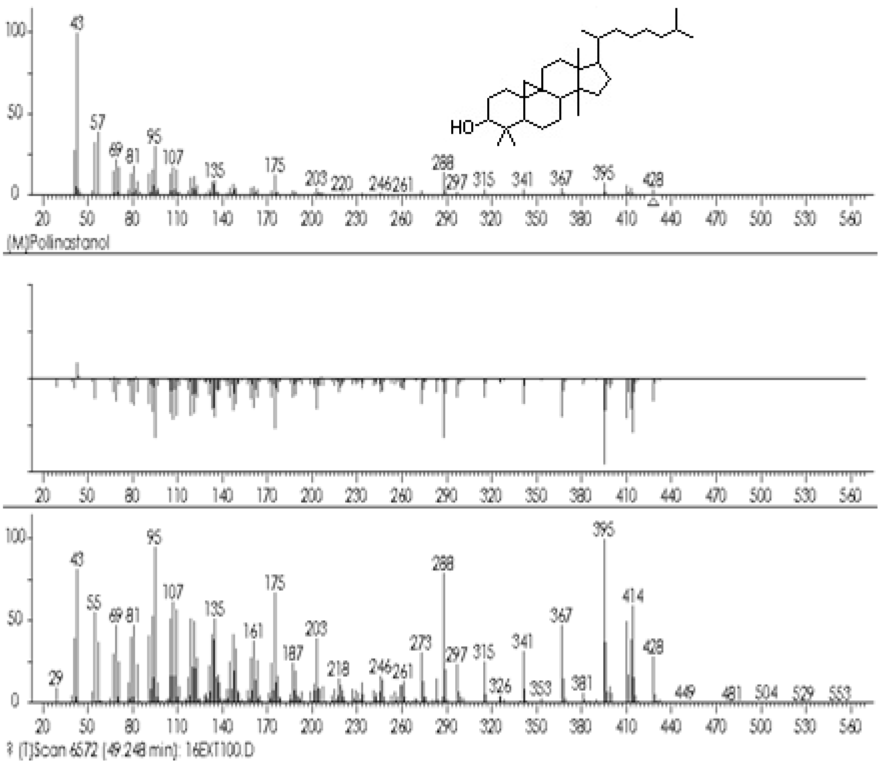
HO
(M)Pollinastanol
(T)Scan 6572 (49.248 min): 16EXT100.D

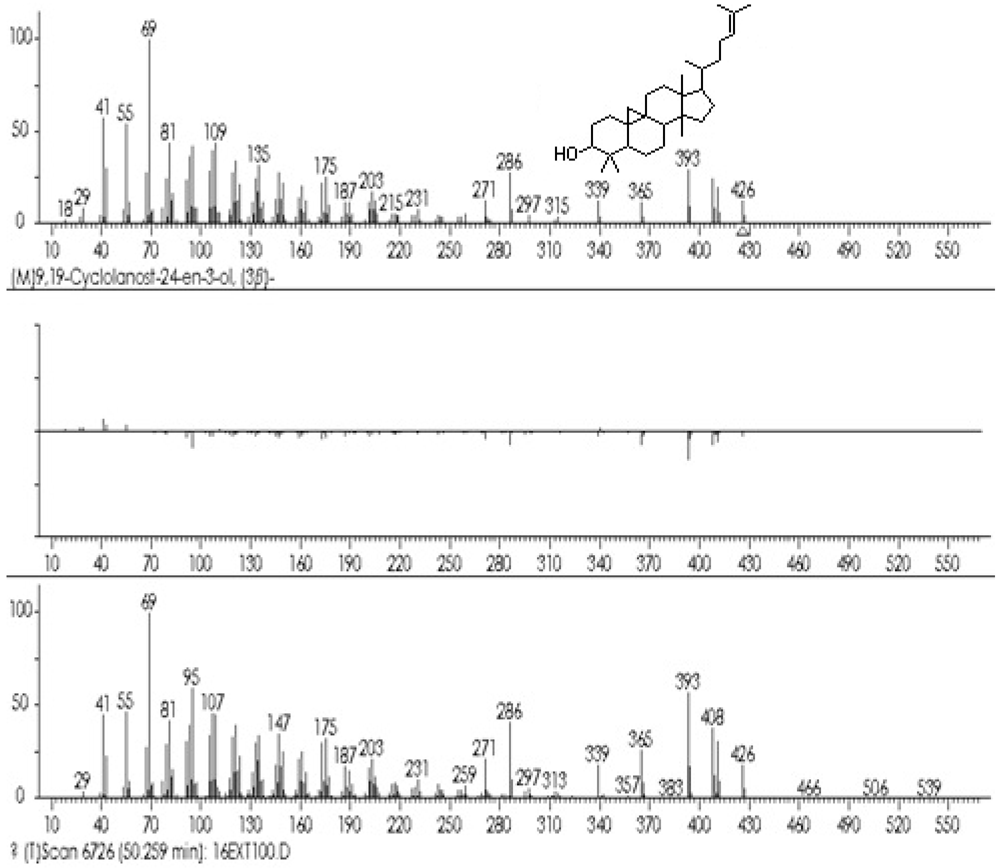

Table 3S . Results of the identification of the compounds by gas chromatography-mass spectrometry. Fraction 53 (F53)

Acq On: 18 Aug 2016 18:19 Sample: F53 Spitless 70°C-1min: 5°C/min-250°C-1min,10°C/min-285°C-20min

peak #	R.T. min	first scan	max scan	last scan	PK TY	peak height	corr. area	corr. % max.	% of total	Compound
1	19.866	2092	2099	2106	M	33135	788193	2.47%	1.242%	
2	36.951	4691	4700	4712	M4	79593	2202292	6.91%	3.471%	(M)Hexadecanoic acid, 2-hydroxy-1-(hydroxymethyl) ethyl ester
3	38.987	4939	5010	5056	M4	160184	31862773	100.00%	50.216%	(M) Isopropyl linoleate
4	39.716	5112	5121	5132	M3	211331	7649320	24.01%	12.055%	(M)9,12-Octadecadienoic acid (Z,Z)-,2.3-dihydroxypropyl ester
5	39.835	5133	5139	5153	M3	297546	9500776	29.82%	14.973%	(M)Nonanoic acid, 9-(3-hexenylidenecyclopropylidene)-,2 -hydroxy-1-(hydroxymethyl) ethyl ester, (Z,Z,Z)-
6	48.564	6446	6468	6489	M5	200279	11448004	35.93%	18.042%	(R) Sitosterol

Sum of corrected areas: 63451358

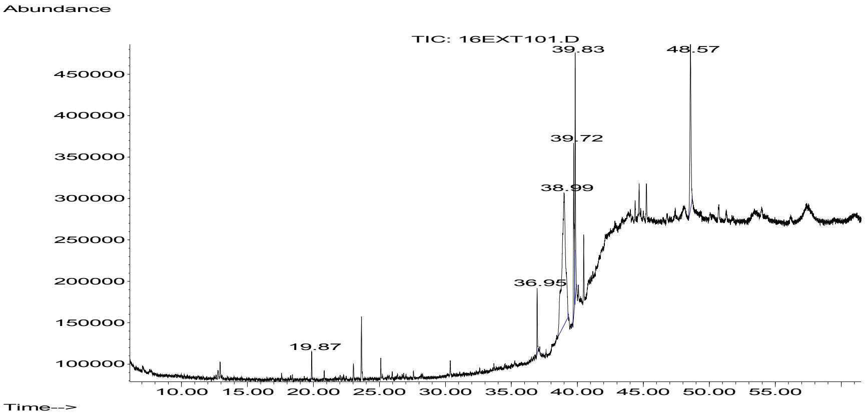
Abundance
TIC: 16EXT101.D
39.83
48.57
39.72
38.99
36.95
19.87
450000
400000
350000
300000
250000
200000
150000
100000
10.00 15.00 20.00 25.00 30.00 35.00 40.00 45.00 50.00 55.00
Time-->

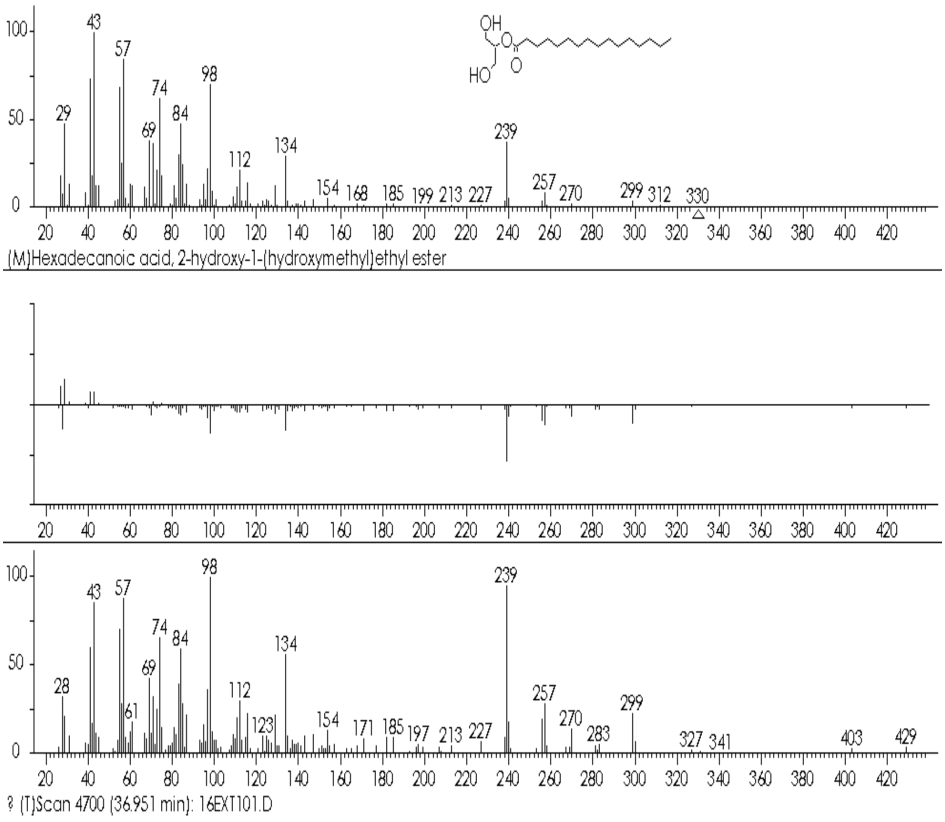
(M)Hexadecanoic acid, 2-hydroxy-1-(hydroxymethyl)ethyl ester
OH
HO
O
29 43 57 69 74 84 98 112 134 154 168 185 199 213 227 239 257 270 299 312 330
28 43 57 61 69 74 84 98 112 123 134 154 171 185 197 213 227 239 257 270 283 299 327 341 403 429
? (T)Scan 4700 (36.951 min): 16EXT101.D

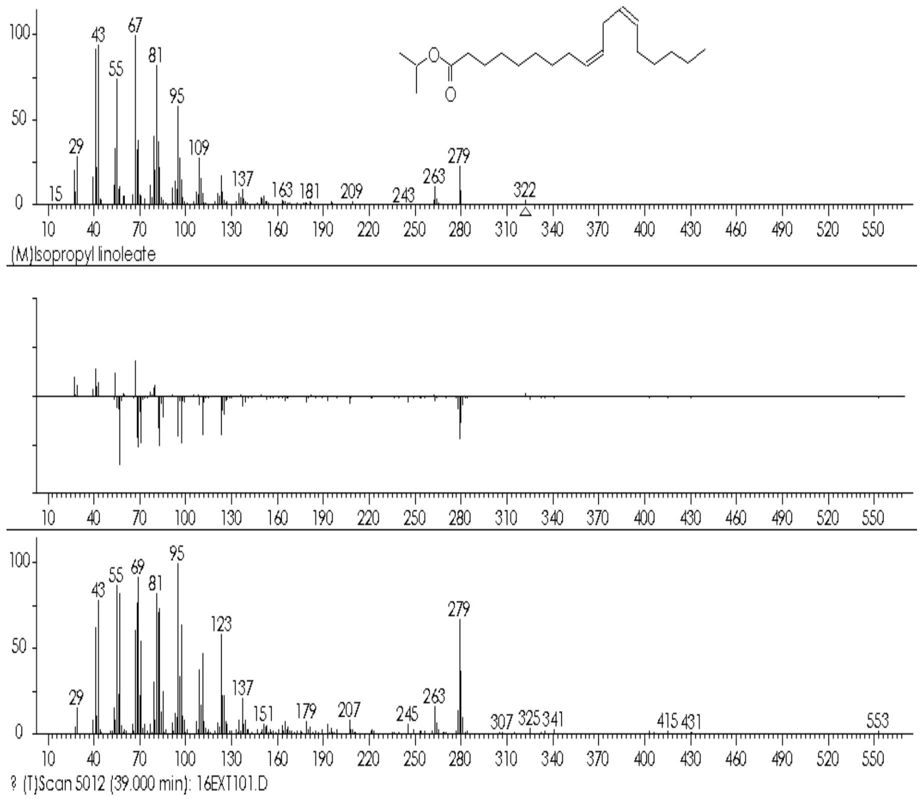
(M)Isopropyl linoleate
? (T)Scan 5012 (39.000 min): 16EXT101.D

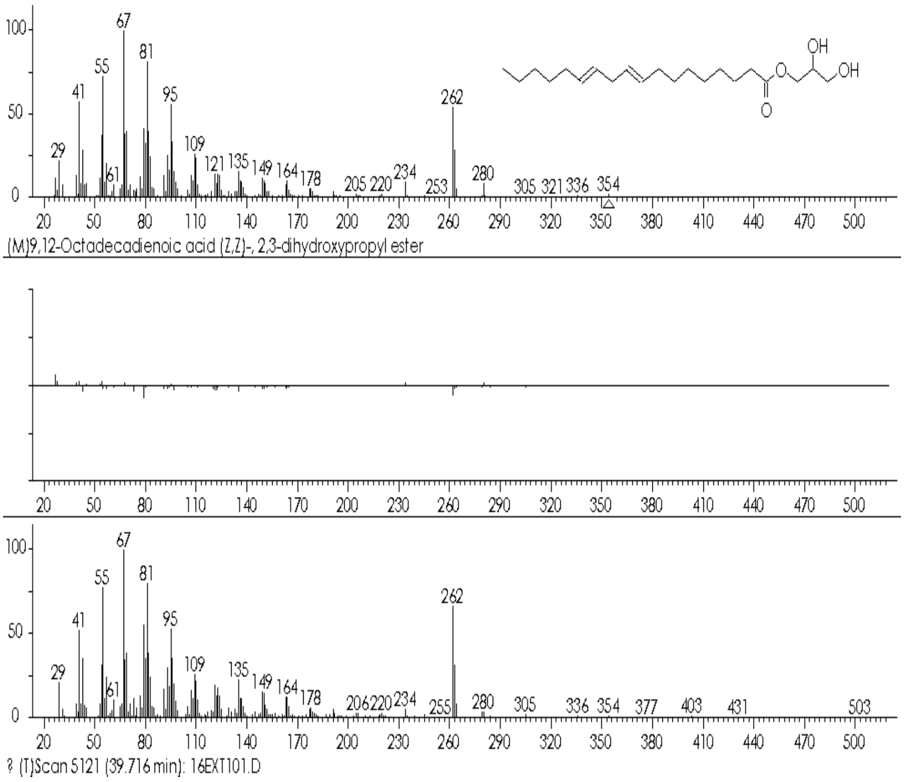
(M)9,12-Octadecadienoic acid (Z,Z)-, 2,3-dihydroxypropyl ester
? (T)Scan 5121 (39.716 min): 16EXT101.D

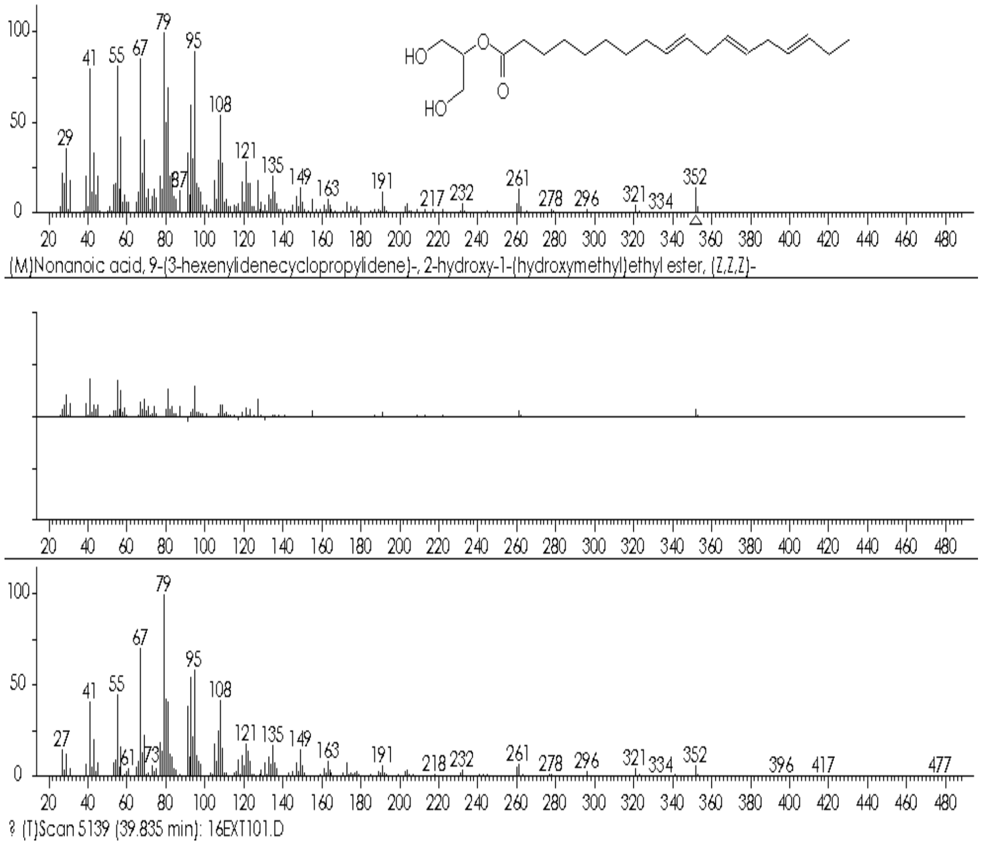
(M)Nonanoic acid, 9-(3-hexenylidenecyclopropylidene)-, 2-hydroxy-1-(hydroxymethyl)ethyl ester, (Z,Z,Z)-
? (T)Scan 5139 (39.835 min): 16EXT101.D

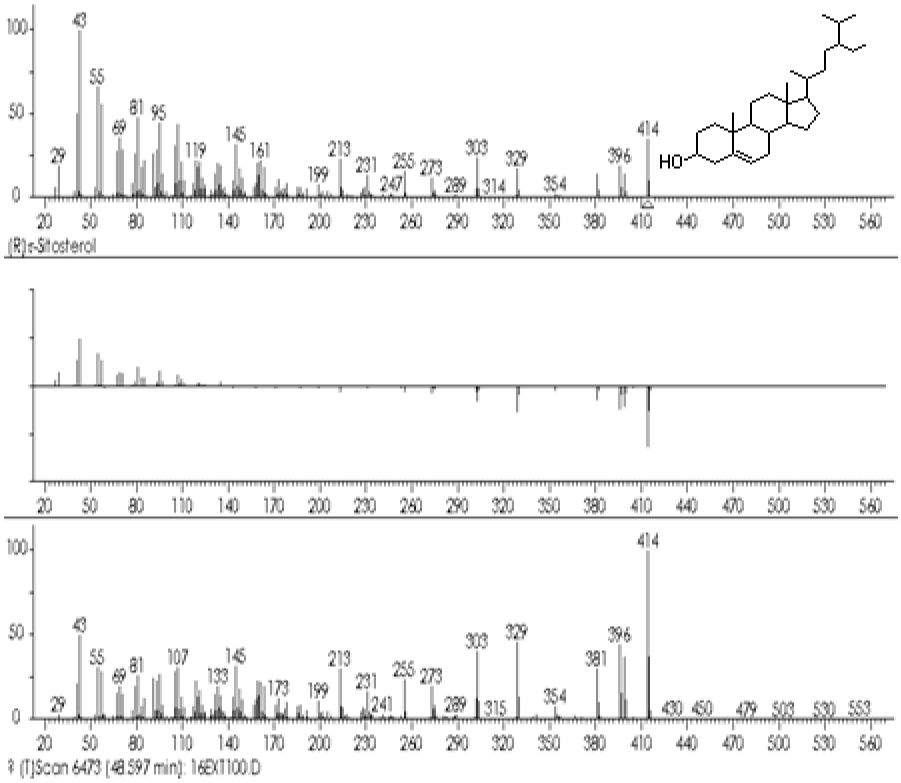
(R)ɛ-Sitosterol
? (T)Scan 6473 (48.597 min): 16EXT100.D

Table 4S . Results of the identification of the compounds by gas chromatography-mass spectrometry.

Fraction 57 (F57)

Acq On: 18 Aug 2016 15:57 Sample: 57 Spitless 70°C-1min, 5°C/min-250°C-1min,10°C/min-285°C-20min

peak #	R.T. min	first scan	max scan	last scan	PK TY	peak height	corr. area	corr. % max.	% of total	Compound
1	7.708	240	248	269	M3	78775	2827602	2.59%	1.774%	(R)Nonal
2	11.642	832	847	866	M2	350935	10032642	9.20%	6.295%	(M)2-Decenal, (E)-
3	14.243	1235	1243	1254	M2	177024	4294856	3.94%	2.695%	(M)2-Undecenal
4	35.250	4433	4441	4450	M2	82199	2197886	2.02%	1.379%	(R)Hexanedioic acid, bis(2-ethylhexyl) ester
5	39.539	4977	5094	5137	M8	417102	109049073	100.00%	68.422%	(M)Isopropyl linoleate
6	45.228	5942	5960	5980	BB2	422566	17806232	16.33%	11.172%	(M)3-(1,5-Dimethyl-hexyl)-3a,10,10,12b-tetramethyl-1,2,3,3a,4,6,8,9,10,10a,11,12,12a,12b-tetradecahydro-benzo[4,5]cyclohepta[1,2-E]inde
7	50.726	6773	6797	6820	M3	204160	13169061	12.08%	8.263%	(M)Stigmasta-3,5-dien-7-one

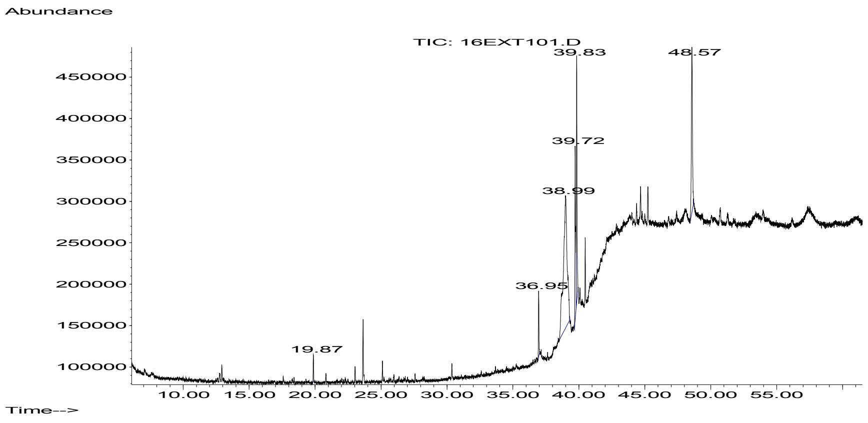

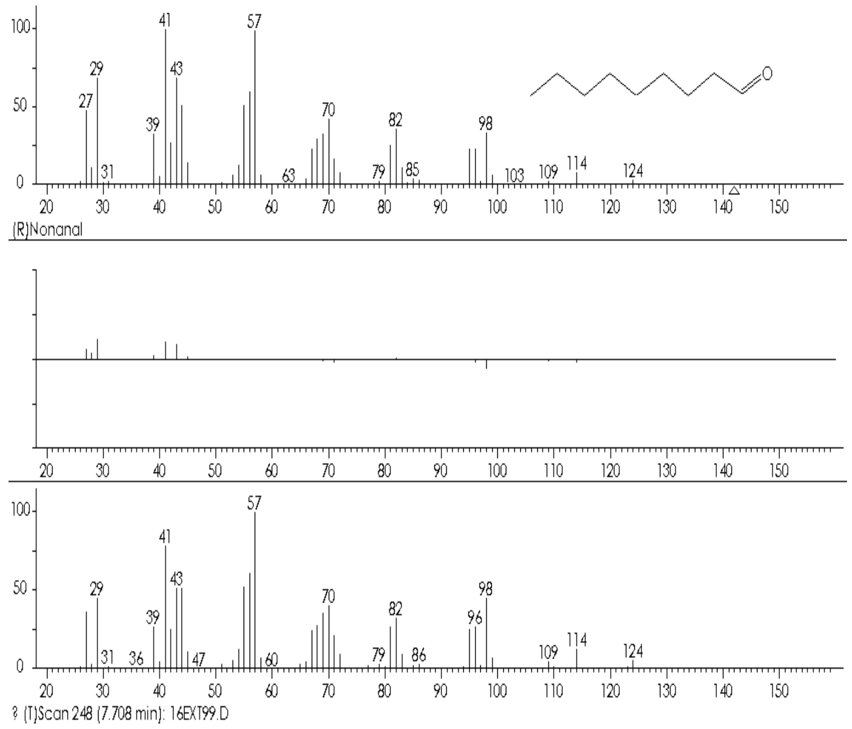
(R)Nonanal
? (T)Scan 248 (7.708 min): 16EXT99.D

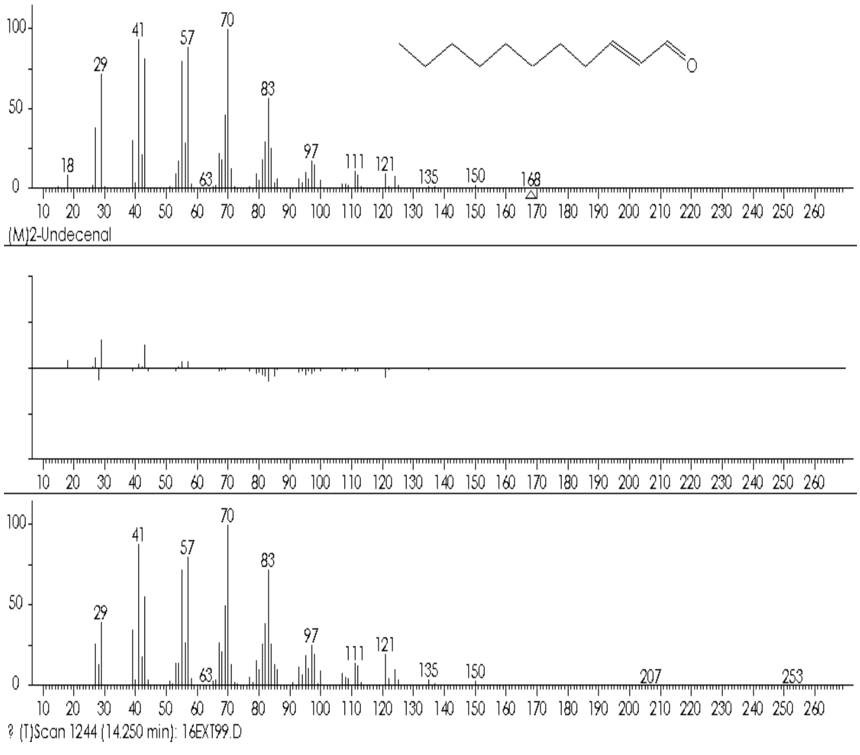
(M)2-Undecenal
? (T)Scan 1244 (14.250 min): 16EXT99.D

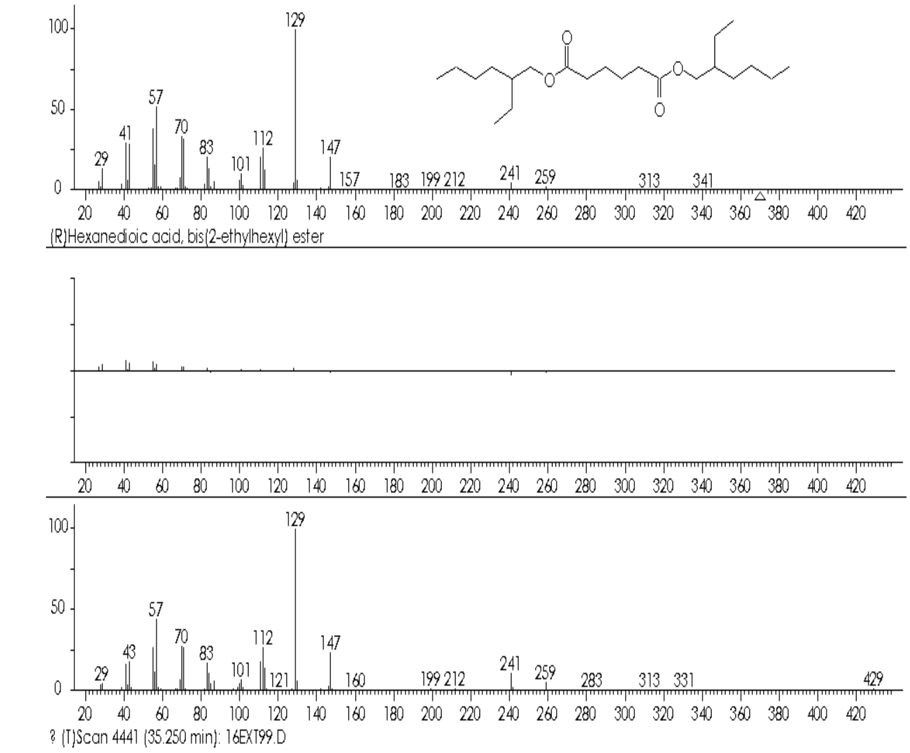
(R)Hexanedioic acid, bis(2-ethylhexyl) ester
? (T)Scan 4441 (35.250 min): 16EXT99.D

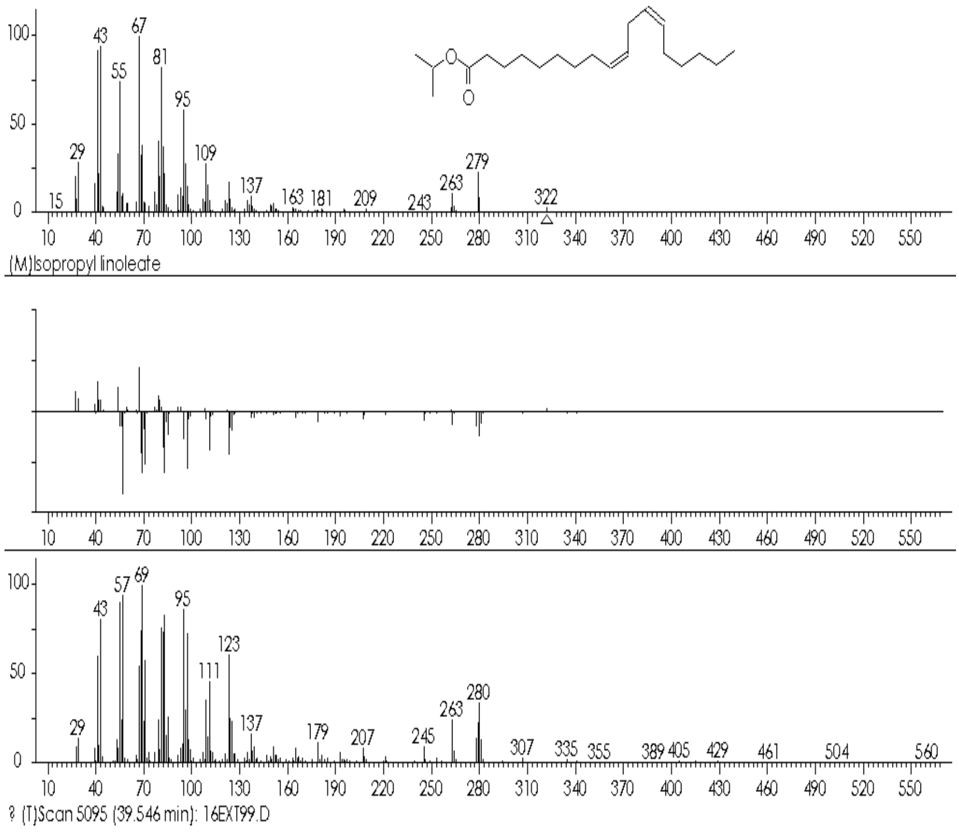
(M)Isopropyl linoleate
? (T)Scan 5095 (39.546 min): 16EXT99.D

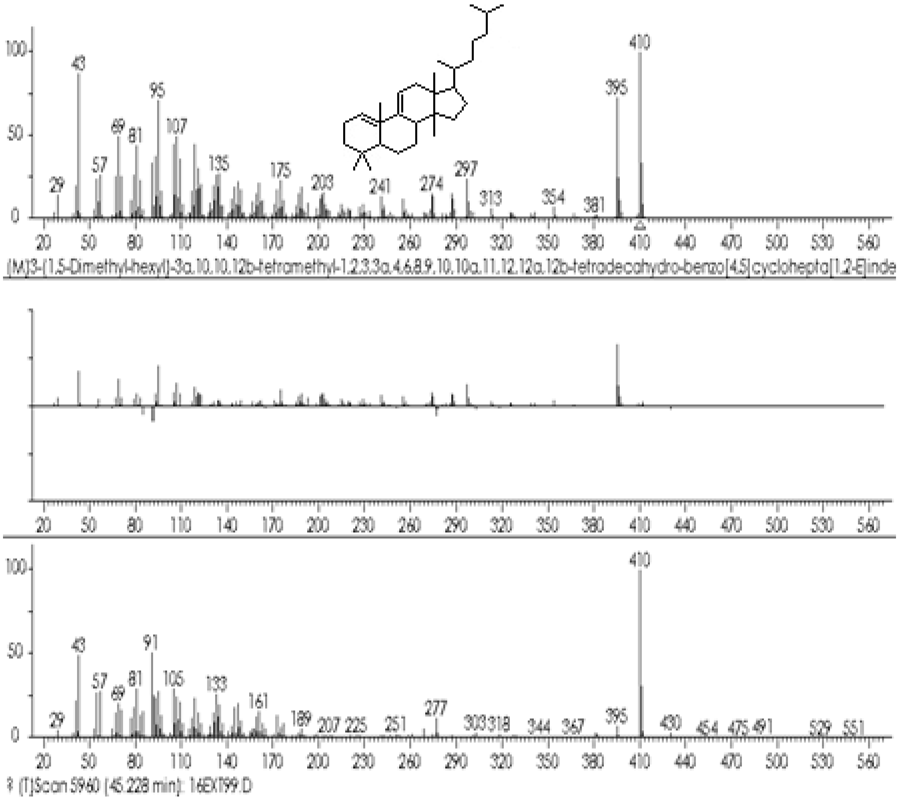
(M)3-(1,5-Dimethyl-hexyl)-3a,10,10,12b-tetramethyl-1,2,3,3a,4,6,8,9,10,10a,11,12,12a,12b-tetradecahydro-benzo[4,5]cyclohepta[1,2-E]inde
(T)Scan 5960 (45.228 min): 16EXT99.D

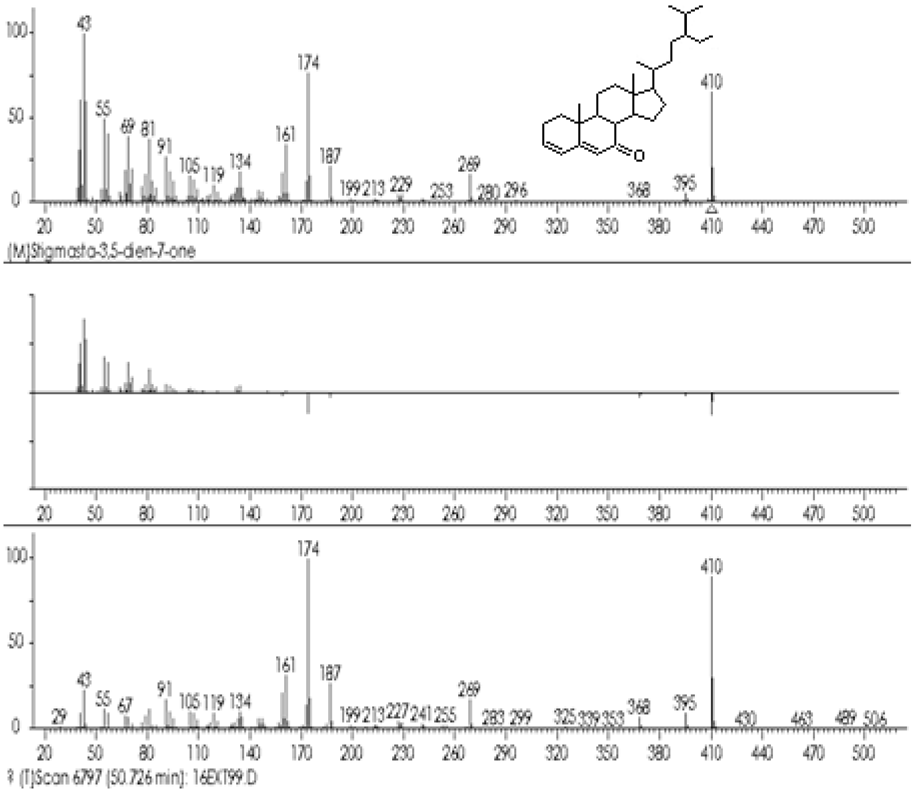
(M)Stigmasta-3,5-dien-7-one
(T)Scan 6797 (50.726 min): 16EXT99.D
